# In vivo clonal tracking reveals evidence of haemangioblast and haematomesoblast contribution to yolk sac haematopoiesis

**DOI:** 10.1038/s41467-022-35744-x

**Published:** 2023-01-03

**Authors:** C. Biben, T. S. Weber, K. S. Potts, J. Choi, D. C. Miles, A. Carmagnac, T. Sargeant, C. A. de Graaf, K. A. Fennell, A. Farley, O. J. Stonehouse, M. A. Dawson, D. J. Hilton, S. H. Naik, S. Taoudi

**Affiliations:** 1 The Walter and Eliza Hall Institute of Medical Research, Melbourne, VIC Australia; 2 Department of Medical Biology, The University of Melbourne, Melbourne, VIC Australia; 3 Peter MacCallum Cancer Centre, Melbourne, VIC Australia; 4 Sir Peter MacCallum Department of Oncology, The University of Melbourne, Melbourne, VIC Australia; 5 The University of Melbourne Centre For Cancer Research, The University of Melbourne, Melbourne, VIC Australia

**Keywords:** Haematopoiesis, Angiogenesis, Cell lineage

## Abstract

During embryogenesis, haematopoietic and endothelial lineages emerge closely in time and space. It is thought that the first blood and endothelium derive from a common clonal ancestor, the haemangioblast. However, investigation of candidate haemangioblasts in vitro revealed the capacity for mesenchymal differentiation, a feature more compatible with an earlier mesodermal precursor. To date, no evidence for an in vivo haemangioblast has been discovered. Using single cell RNA-Sequencing and in vivo cellular barcoding, we have unravelled the ancestral relationships that give rise to the haematopoietic lineages of the yolk sac, the endothelium, and the mesenchyme. We show that the mesodermal derivatives of the yolk sac are produced by three distinct precursors with dual-lineage outcomes: the haemangioblast, the mesenchymoangioblast, and a previously undescribed cell type: the haematomesoblast. Between E5.5 and E7.5, this trio of precursors seeds haematopoietic, endothelial, and mesenchymal trajectories.

## Abstract (web summary)

The lineage relationship between blood and endothelial cells has been difficult to examine due to the multiphasic timing of hematopoiesis in the embryo. Here the authors use using in vivo barcoding technology to assess cell ancestry and show that blood and endothelial cells emerge through common (haemangioblast) or separate (mesenchymoangioblasts and haematomesoblasts) progenitors in the yolk sac.

## Introduction

Haematopoiesis first occurs in the embryonic day (E) 7.0–E10.5 mouse yolk sac to produce the mature haematopoietic lineages (primitive erythrocytes, megakaryocytes, macrophages)^1–4^ and erythro-myeloid progenitors^2,5^. Our understanding of how this occurs is predominantly informed by in vitro and ex vivo data that have suggested a differentiation sequence involving mesoderm, the haemangioblast (a precursor that gives rise to both haematopoietic and endothelial lineages), and the haemogenic endothelium (a precursor defined by dual haematopoietic and endothelial marker expression but committed to the blood lineage)^6–12^. A long-standing question has been whether the yolk sac-derived endothelial and haematopoietic lineages share a common clonal origin independent from local mesenchymal lineages (smooth muscle, fibroblast, mesothelium). The balance of in vitro evidence suggests that although cells from the gastrulating embryo are capable of generating smooth muscle, endothelium, and haematopoietic lineages in vitro^10^, dual endothelial-haematopoietic outcome is a rare occurrence from the extraembryonic mesoderm and haemogenic endothelium^7,8,13^. Lineage tracking studies suggest that dual endothelial-haematopoietic outcome may occur at a higher frequency^14^ in vivo, although mesenchymal contribution was not addressed in this study.

Embryonic blood cells are classified with reference to their ancestry. Cells derived directly from the mesoderm without transiting through haematopoietic stem cells or multipotent erythro-myeloid progenitors (EMPs) are termed primitive. Haematopoietic cells that descend from a bona fide EMP or a stem cell are considered to be pro-definitive and definitive, respectively. Haematopoietic stem cells emerge between embryonic day (E) 10.5–E11.5^15,16^, therefore haematopoietic lineages that emerge prior to E11.5 must derive directly from the mesoderm or from EMPs. Yolk sac primitive erythrocytes and megakaryocytes can derive from a common precursor^3^, have features that distinguish them from their stem cell-derived counterparts^4,17,18^, and are generated in the absence of EMPs^4,19–21^. Accordingly, both lineages have been proposed to be primitive lineages. In contrast, yolk sac EMP-derived macrophages are largely indistinguishable from those derived from stem cells^22,23^. This, combined with the observation that fetal macrophages are not produced in the absence of EMPs^21^, suggests that macrophages are not a primitive lineage. Although the primitive-definitive classification convention is based on sound deductive reasoning, whether it accurately predicts the in vivo ancestral relationship between the yolk sac haematopoietic lineages remains untested.

To understand the in vivo cellular genealogy of the first mesodermal derivatives, we investigated the yolk sac between  E7.25–E10.5 using single-cell transcriptomics and between E5.5-E10.5 using single cell lineage tracking by in vivo barcoding. Herein, we provide in vivo evidence of the haemangioblast. We also show that haemangioblasts are not the sole blood and endothelial precursor. Rather, these lineages arise from a trio of progenitors with distinct patterns of lineage production: the haemangioblast (that produces haematopoietic lineages and conventional endothelium), the mesenchymoangioblast (that produces mesenchyme and conventional endothelium), and the haematomesoblast which is a previously undescribed class of haematogenic precursor that that produces haematopoietic lineages and mesenchyme, and so bridges the haematopoietic and mesenchymal elements of the yolk sac.

## Results

### A putative mesenchymal axis of yolk sac haematopoiesis

**Fig. 1:**
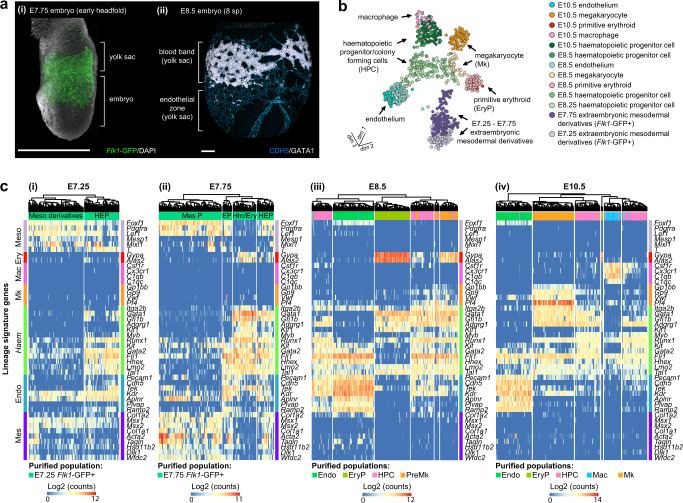
Single-cell transcriptomics profiling of the early endothelial-haematopoietic landscape. **a** (i) Distribution of *Flk1*-GFP-expressing extraembryonic mesodermal derivatives at E7.75 (*n* = 7 embryos); (ii) Distribution of haematopoietic cells (GATA1+, white) and endothelium (GATA1− CDH5+, blue) at E8.5 (*n* = 12 embryos). Scale bars, 300 μm. **b** UMAP dimension reduction representation of 926 transcriptomes from cells collected from the yolk sac between E7.25–E10.5. **c** (i) Heatmap of key mesodermal, haematopoietic, endothelial, and mesenchymal genes expression in E7.25 to E10.5 yolk sac populations. (i) E7.25, (ii) E7.75, (iii) E8.5, (iv) E10.5. Endo: endothelium, EP endothelial precursors, Ery erythrocyte, EryP primitive erythrocyte, HEP haematoendothelial progenitors, haematopoietic progenitor/colony forming cells (HPC), Mac macrophage, Mk megakaryocyte, Mes mesenchyme, Meso derivatives mesodermal derivatives, Mes P mesenchymal precursors.

The extraembryonic mesoderm of the E7.75 yolk sac lines the primitive endoderm as a sheet^1^ (Fig. 1ai). By E8.5, this sheet has become morphologically and immunophenotypically diversified into mesenchymal cells^1^, the blood band (which contains a mix of primitive erythroid cells, megakaryocyte precursors, EMPs, endothelium, and mesothelial cells)^24–26^, and the endothelial zone^24^ (Fig. 1aii). The E8.5 yolk sac also contains three immunophenotypically identifiable haematopoietic lineages (primitive erythrocyte, megakaryocyte, and haematopoietic progenitor/colony forming cells (HPC) (a population containing all EMP activity, Supplementary Fig. 1)^4^ and the endothelium^4,24,27,28^ (Supplementary Fig. 2a–d). Although macrophage-associated genes can be detected at E8.5^29^, significant numbers of bona fide macrophages are not detected before E10.5 (Supplementary Fig. 2e)^4^.

To define the transcriptional identity of cellular intermediates that appear during mesodermal diversification in the yolk sac, we generated a targeted single-cell RNA-sequencing (scRNA-Seq) dataset encompassing all known cellular immunophenotypes associated with endothelial and haematopoietic differentiation in the yolk sac between E7.25–E10.5 (Supplementary Data 1 and 2). Using the gating strategies described in Supplementary Fig. 2a–e and the immunophenotypes listed in Supplementary Data 2, we collected:

- E7.25 and E7.75 *Flk1*-expressing extraembryonic mesodermal derivatives, the source of haematopoietic and endothelial cells^30,31^.
- E10.5 endothelium, primitive erythrocyte, megakaryocyte, HPC (Supplementary Fig. 2e)^4,18^ and macrophage lineages, which served as endpoint references.
- To understand developmental transitions: early primitive erythroid cells, megakaryocytes (Supplementary Fig. 2c, d)^4^, and endothelial cells were collected at E8.5; HPCs were collected at E8.25, E8.5, and E9.5 (Supplementary Fig. 2c–e).

Collected transcriptomes of 926 cells (out of 1073 captured) were compiled to generate a 3D Uniform Manifold Approximation and Projection (UMAP) of the E7.25–E10.5 yolk sac lineages (Fig. 1b, Supplementary Fig. 2f, Supplementary Data 3, and Supplementary Software 1 and 2). This revealed that from E8.5, endothelium and blood lineages were readily identifiable, and that E7.25 and E7.75 populations had a more ambiguous identity, which is consistent with cells being in a state of developmental transition. Of note, a caveat of this approach is that the same broad cell type (e.g., EryP) could have different transcriptional features at different developmental stages.

To follow mesoderm differentiation into blood, endothelium, and mesenchyme we investigated the evolution of transcriptional signatures for mesoderm (*Foxf1, Pdgfra, Lef1, Mesp1, Mixl*, and *T*), erythroid (*Gypa and Alas2*), macrophage (*Csf1r, Cx3cr1, C1qb*, and *C1qc*), megakaryocyte (*Gp1bb, Gp9, Vwf*, and *Pf4*), haematopoietic (*Itga2b, Gata1, Gfi1b, Adgrg1, Klf1, Myb, Runx1, Kit, Gata2, Fli1, Hhex, Lmo2*, and *Tal*1), endothelial (*Pecam1, Cdh5, Tek, Kdr, Aplnr, Pvlap*, and *Ramp2*) and mesenchymal (*Col1a2, Msx1, Msx2, Col1a1, Acta2, Tagln, Hsd11b2, Dlk1*, and *Wfdc2*) identities between E7.25 and E10.5 (Fig. 1ci–iv). Transcriptional identities correlated well with immunophenotypically defined populations^4^ (Supplementary Data 2) at E10.5 (Fig. 1civ, Supplementary Fig. 3a, b):

- Endothelium (TER119− CD45− CD41− CDH5+ cells) were characterised by *Pecam1*, *Cdh5*, *Tek*, *Kdr*, *Aplnr*, *Plvap* and *Ramp2* expression.

- Primitive erythroid cells (TER119+ CD41− CD45− CDH5−) expressed *Gypa* and *Alas2*.
- Megakaryocytes (TER119− CDH5− CD41+ CD45− cells) expressed *Gp1bb*, *Gp9*, *Vwf*, *Pf4*, and *Itga2b*.
- Macrophages (TER119− CD45+ CD41− cells) expressed *Cx3cr1*, *Csf1r*, *C1qb*, and *C1qc*.
- HPCs (TER119− CDH5− CD41low CD45+) were characterised by low/no expression of differentiation markers and robust detection of *Gata1*, *Gfi1b*, *Myb*, *Runx1*, *Kit*, *Gata2*, *Lmo2*, *Tal1*.

Between E7.25 and E8.5, markers that are specific to the endothelium at later developmental stages (*Pecam1, Cdh5, Tek,* and *Kdr*) were also detected in early haematopoietic lineages (Fig. 1ci–iii). At E7.25, a cluster with dual endothelial-haematopoietic markers was readily detectable (haematoendothelial precursors, Fig. 1ci). At E7.75, the *Kit*^*+*^ *Gata2*^*+*^ *Fli1*^*+*^ *Hhex*^*+*^ *Lmo2*^*+*^ *Tal1*^*+*^ haemato-endothelial populations could be segregated into three clusters: erythroid (*Gypa*^*+*^ *Alas2*^*+*^), haematopoietic/endothelial (*Gypa*^*low/−*^ *Alas2*^*low/−*^), and endothelial precursor^29^ (*Myb*^*−*^ *Gata1*^*−*^ *Ramp2*+ *Pecam1*+ *Cdh5*+ *Tek*+ cluster). These findings are consistent with the transcriptional patterns observed by others^8,29,32,33^.

Although haemato-endothelial precursors were transcriptionally distinct from the mesoderm at E7.25, they still exhibited a robust mesenchymal signature at E7.75 (similar to that of non-haemato-endothelial populations) that was abruptly downregulated by E8.5 (Fig. 1ci–iii). In the light of the haemangioblast theory, that postulates an early separation of these lineages^6,7,10–12^, a transcriptional connection between the endothelial, haematopoietic, and mesenchymal lineages was unexpected. Importantly, this suggested that mesenchymal and haemato-endothelial fates might segregate later than thought. Possibly via an as-yet undiscovered common clonal ancestor.

### Tracking mesodermal diversification using cellular barcoding

**Fig. 2:**
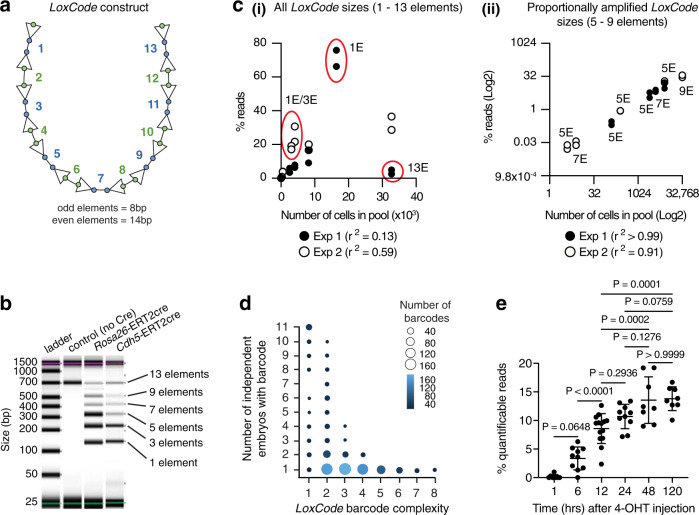
In vivo cellular barcoding using the *LoxCode* mouse line. **a** Schematic of the *LoxCode* construct: LoxP sites (arrowheads), 1–13: unique DNA sequences (elements). *LoxCode* construct size = 616 nucleotides. **b** Representative examples of *LoxCode* construct PCR products before and after recombination with *Rosa26* or *Cdh5* driven ERT2cre recombinase. Number of elements per fragment is indicated. Data is representative of *n* = 104 independent libraries prepared from crosses with *Cdh5*ERT2Cre mice and *n* = 180 independent libraries prepared from crosses with *Rosa26*ERT2Cre mice. **c** Proportionality of barcode amplification in control pools. (i) 1 element and 13 element barcodes are over- and under- represented respectively. (ii) 5–9 element barcode sequences are within a near-linear manner, allowing for reliable quantification of relative clonal output to all assessed lineages (biomass). Note, multiple representation of 1 and 5 element (E) barcodes reflects the presence of multiple independent 1 and 5 element barcode samples in the experiment. Data shown derive from two independent experiments that were performed in technical duplicate. All data points are shown. *r*^2^ represents Pearson’s correlation coefficient for each independent experiment. **d** Complex barcodes (requiring more recombination steps) are rarer and therefore more likely to be clonal. Number of embryos in which barcodes of a given complexity (minimal number of recombination steps) are detected. *n* = 11 independent embryos. **e** % of quantifiable (complex 5–9 element) barcodes after 4-OHT injections. 1 h: 8 embryos; 6 h: 10 embryos, 12 h: 14 embryos, 24 h: 10 embryos, 48 h: 8 embryos, 120 h: 8 embryos. Data were analysed using One-way ANOVA (using Tukey’s *P* value adjustment) was used for multiple comparisons. Exact *p* values are shown. Bars represent mean ± SD.

To test the hypothesis that haemato-endothelial development might occur via mesenchymal as well as endothelial intermediaries, we performed in vivo lineage tracking using inducible cellular barcoding. This approach enables the fate of large numbers of individual cells to be tracked via indelible DNA tagging under physiological conditions^34–37^. Identification of the same DNA tag (or barcode) in two cells, or cell populations, demonstrates that they share a clonal ancestry. To enable the sensitive recovery of barcodes from small numbers of purified cells, we used a Cre-LoxP-based in situ barcoding mouse line (named the *LoxCode* line) that can generate a high diversity of cell-specific barcodes^38^ following Cre exposure during unperturbed development (Fig. 2a, Supplementary Fig. 4a, b, and *Methods*). The *LoxCode* construct contains 14 LoxP sites in alternate orientation interspersed with 13 × 8–14 bp unique DNA segments (termed elements) (Fig. 2a and Supplementary Fig. 4a, b). The theoretical diversity provided by the recombination of the *LoxCode* is >30 × 10^9^ unique barcodes (see *Methods*). A Sanger-sequence verified *LoxCode* cassette was introduced in mice at the *Gt(ROSA)26Sor* locus (*Rosa26*) using CRISPR technology. Exposure to Cre recombinase led to recombination (inversion/deletion) and the expected formation of *LoxCode* cassettes composed of 13, 9, 7, 5, 3, or 1 element(s) (Fig. 2b). Construction of the *LoxCode* line will be described in greater detail elsewhere^39^.

To assess the sensitivity and linearity of barcode detection, control experiments were performed with barcoded *LoxCode*/*Rosa26*CreERT2 acute myeloid leukaemia clones. After in vitro exposure to 4-Hydroxytamoxifen (4-OHT), single acute myeloid leukaemia cells were sorted into individual wells and expanded in vitro yielding clonal lines that were sequenced for barcode identification and pooled in known proportions to assess the sensitivity and linearity of barcode detection in a pool. We found that *LoxCode* sequences between 5 and 9 elements were detected in a near-linear manner (Fig. 2c), providing the potential for reliable quantification of the magnitude of clonal contribution to any lineage (biomass).

*LoxCode* recombination generates a range of barcodes via one to 15 recombination steps (this is referred to as barcode complexity, see *Methods*). High complexity barcodes are detected in few embryos, suggesting a high likelihood of being clonal (Fig. 2d). In contrast, low complexity barcodes are frequently detected in independent embryos, this suggests that they were made independently in several cells and therefore could not be used for clonal tracking. Our filtering steps involved the selection of infrequently occurring and complex barcodes to ensure clonal tracking (Supplementary Figs. 4c, 5, and *Methods*). We found that the limit of sensitivity of barcode detection was approximately 1 in 16,000 cells (Supplementary Data 4).

To investigate the temporal dynamics of complex barcode generation after 4-OHT injection, we induced barcoding at E6.5 and collected embryos 1, 6, 12, 24, 48, or 120 h later. Complex barcodes were detected as early as 1 h after induction and steadily accumulated over the first 24 h (Fig. 2e). After 24 h the proportion of quantifiable barcodes reads was stable (Fig. 2e). Thus, when barcodes are generated during lineage diversification, developmental intermediaries can be labelled. This enables reconstruction of in vivo cellular genealogies.

### Benchmarking the *LoxCode* mouse for yolk sac lineage tracking

To benchmark the *LoxCode* mouse line in the yolk sac, we first performed control experiments involving lineages that were known to be separate (negative control: primitive erythrocytes and non-erythroid haematopoietic lineages^40,41^), or known to be connected (positive control: yolk sac macrophages and brain macrophages [microglia]^23,42,43^).

**Fig. 3:**
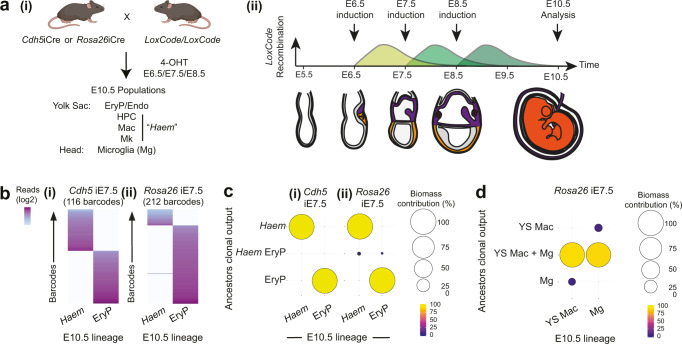
Benchmarking *LoxCode* barcoding in the yolk sac. **a** (i) Experimental design for barcoding experiments. Experimental mice received only one dose of 4-OHT (at either E6.5, E7.5, or E8.5). Created with BioRender.com. (ii) Barcoding window in reference to 4-OHT injection time. **b** Primitive erythrocytes/*Haem* relationships after *Cdh5*iCre induction at E7.5 ((i), 116 informative barcodes, *n* = 5 embryos) and *Rosa26*iCre induction at E7.5 ((ii), 212 informative barcodes, *n* = 3 embryos). **c** Biomass analysis using 5–9 elements complex barcodes of primitive erythrocytes/*Haem* relationships after *Cdh5*iCre ((i), 111 barcodes, *n* = 5 embryos) or *Rosa26*iCre ((ii), 211 barcodes, *n* = 3 embryos) induction at E7.5. **d** Biomass analysis of yolk sac (YS) and head macrophage (microglia (Mg)) relationships after *Rosa26*iCre induction at E7.5. 49 barcodes, *n* = 3 embryos.

We used inducible Cre lines that would either label all cells (*Rosa26*-ERT2-Cre, referred to as *Rosa*iCre) or would label *Cdh5*-expressing cells (*Cdh5*-ERT2-Cre^44^, referred to as *Cdh5*iCre). At early developmental stages (E6.5–E8.5), *Cdh5*-expressing cells include precursors to the endothelial and haematopoietic lineages (E6.5–E8.5), and at E8.5 the conventional endothelium itself (refs. 28, 29, 32, 45, 46 and Fig. 1c and Supplementary Fig. 2f). Barcode labelling was induced with 4-OHT between E6.5–E8.5 and offspring of barcoded cells were analysed in the E10.5 yolk sac lineages (Fig. 3a and Supplementary Fig. 4d). After collection of E10.5 yolk sac lineages by flow cytometry (Supplementary Data 5), *LoxCode* libraries were generated, sequenced and analysed following the pipeline described in Supplementary Fig. 5 and *Methods*. In the negative control experiment, we found that primitive erythrocyte and the non-erythroid haematopoietic lineages (HPC, macrophage, and megakaryocyte lineages, referred to herein as the *Haem* group) were on separate trajectories from E7.5 (Fig. 3b, c and Supplementary Fig. 6a–c). This was consistent with population-level lineage tracking (Supplementary Fig. 6d), the early segregation of primitive erythrocytes inferred by our scRNA-Seq data (Fig. 1b, 1cii and Supplementary Fig. 2f), the independence of the primitive erythrocytes from the HPCs^40,41^, and the in vivo divergence of Mk and EryP lineage recently described^47^. This suggested that the *LoxCode* molecular protocol and analysis pipeline did not create spurious connections. In the positive control experiment, we found that >90% of yolk sac macrophages and cephalic microglia populations shared clonal ancestors at E7.5 (Fig. 3d). This demonstrated that expected ancestries were robustly detected using our method.

**Fig. 4:**
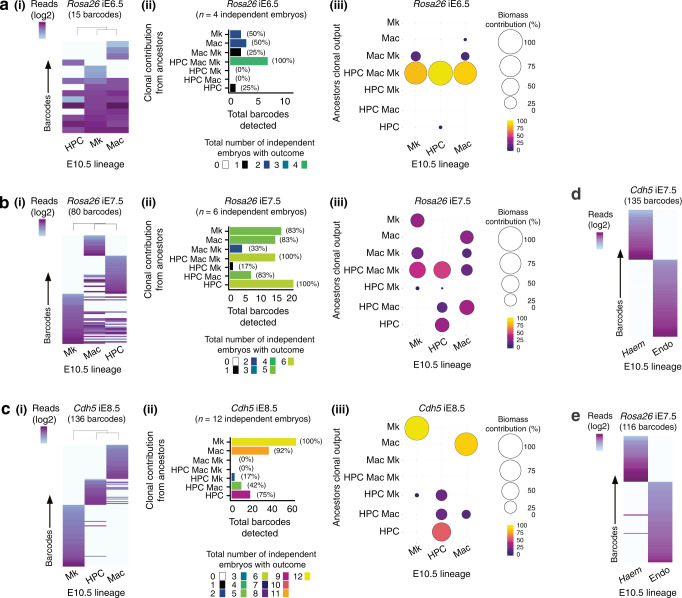
Investigating haematopoietic and endothelial lineage relationships. **a** Induction of barcode formation at E6.5 using *Rosa26*iCre (15 barcodes, *n* = 4 embryos). (i) Heatmaps of all informative barcodes. (ii) Biological reproducibility of clonal outcomes. Colours represent the total number of independent embryos with the stated clonal outcome. Values in parentheses represent the percentage of independent embryos in which the clonal outcome was observed. (iii) Summary of contribution to the E10.5 yolk sac non-erythroid haematopoietic lineages biomass (based on 5–9 element barcodes [12 barcodes]). **b** Induction of barcode formation at E7.5 using *Rosa26*iCre (80 barcodes, *n* = 6 embryos). (i) Heatmaps of all informative barcodes. (ii) Biological reproducibility of clonal outcomes. Colours represent the total number of independent embryos with the stated clonal outcome. Values in parentheses represent the percentage of independent embryos in which the clonal outcome was observed. (iii) Summary of contribution to the E10.5 yolk sac non-erythroid haematopoietic lineages biomass (based on 5–9 element barcodes [77 barcodes]). **c** Induction of barcode formation at E8.5 induction using *Cdh5*iCre (136 barcodes, *n* = 12 embryos). (i) Heatmaps of all informative barcodes. (ii) Biological reproducibility of clonal outcomes. Colours represent the total number of independent embryos with the stated clonal outcome. Values in parentheses represent the percentage of independent embryos in which the clonal outcome was observed. (iii) Summary of contribution to the E10.5 yolk sac non-erythroid haematopoietic lineages biomass (based on 5–9 element barcodes [109 barcodes]). **d** Barcode distribution between the E10.5 yolk sac endothelial (Endo) and non-erythroid haematopoietic (*Haem*) lineages after *Cdh5*iCre induction at E7.5. 135 informative barcodes, *n* = 6 independent embryos. **e** Barcode distribution between the E10.5 yolk sac endothelial (Endo) and non-erythroid haematopoietic (*Haem*) lineages after *Rosa26*iCre induction at E7.5. 116 informative barcodes, *n* = 3 independent embryos.

In addition, we found that the *Haem* sub-lineages derived from common clonal ancestors at E6.5 and seeded independent macrophage, HPC, and megakaryocyte trajectories between E7.5 and E8.5 (Fig. 4a–c and Supplementary Fig. 7). This again demonstrated the robustness and specificity of detected connection with this method of in vivo cellular barcoding.

### Endothelial and haematopoietic cells diverge from E7.5

We next investigated the relationship between the *Haem* group and the endothelium. To this end, barcode formation was induced in E7.5 and E8.5 *Cdh5*-expressing cells using *LoxCode*:*Cdh5*iCre mice, and then recovered in the E10.5 yolk sac. This revealed that *Cdh5*-expressing clones contributed to either *Haem* cells or endothelium but not to both lineages (Fig. 4d and Supplementary Fig. 8a, c). We used *LoxCode:Rosa26*iCre mice to enable unbiased labelling, this largely confirmed that the endothelium and *Haem* group were ancestrally independent. However, from 116 clones two instances of dual lineage contribution were observed (Fig. 4e and Supplementary Fig. 8b). Although rare and only a minor contributor to the E10.5 biomass, this pattern of contribution was consistent with the haemangioblast theory.

### Discovery of the haematomesoblast

**Fig. 5:**
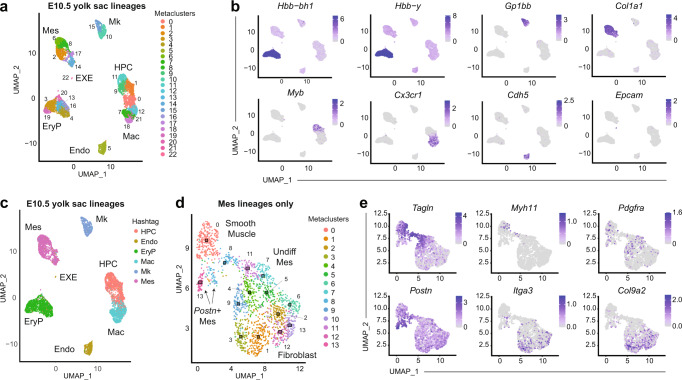
Characterisation of all E10.5 yolk sac mesoderm derivatives. **a** Seurat clustering of single cell RNAseq of yolk sac mesodermal populations: EryP (TER119+CD45−), Mk (TER119− CD41+ CD45−), HPC (TER119− CD41+ CD45+), Mac (TER119− CD41− CD45+), Endo (TER119− CD41− CD45− CD31+), Mes (TER119− CD41− CD45− CD31−). EXE, extraembryonic endoderm. **b** Representative marker genes for each of the metaclusters identified on (**a**). **c** Correspondance between population of origin and transcriptional identity in scRNA-Seq of yolk sac populations. Each population was labelled with an individual barcode (hashtag) before processing. Transcriptional identity is indicated (labels near metaclusters). Hashtag calls show good correspondence with immunophenotype. **d** Clustering of Mes populations reveals immature but distinct populations. Mes: mesenchyme. Undiff: undifferentiated. **e** Representative marker genes for the populations identified on (**d**) Endo (endothelium), EryP (primitive erythroid), Mk (megakaryocyte), HPC (haematopoietic progenitor/colony forming cell), Mac (macrophage), EXE extraembryonic endoderm.

To define all the mesodermal derivatives present in the yolk sac, we purified E10.5 endothelium, blood lineages, and TER119- CD41- CD45- CD31- mesodermal derivatives (collectively termed mesenchyme) for 10X Genomics single cell RNA sequencing. From 7316 high quality single cells, seven transcriptional metaclusters were identified: primitive erythroid, megakaryocyte, HPC, macrophage, endothelium, mesenchyme and a handful of extraembryonic endoderm cells (Fig. 5a, b, Supplementary Data 6). As we observed with the E7.25–E10.5 yolk sac scRNA-Seq dataset (Fig. 1c), there was a good correlation between transcriptional identity and immunophenotype (Fig. 5a–c, Supplementary Data 6, and Supplementary Fig. 3c, d). Within the mesenchymal cluster four main populations could be recognised (Fig. 5d–e and Supplementary Fig. 9a, b). All mesenchyme clusters displaying a robust mesenchymal signature which included: (1) immature smooth muscle cells (Supplementary Fig. 9c); (2) fibroblasts (Supplementary Fig. 9d); (3) undifferentiated mesenchyme (Supplementary Fig. 9e); and, (4) and two small clusters with high *Postn* expression (Supplementary Fig. 9f). Additional marker genes for each of these clusters can be found in Supplementary Data 7.

**Fig. 6:**
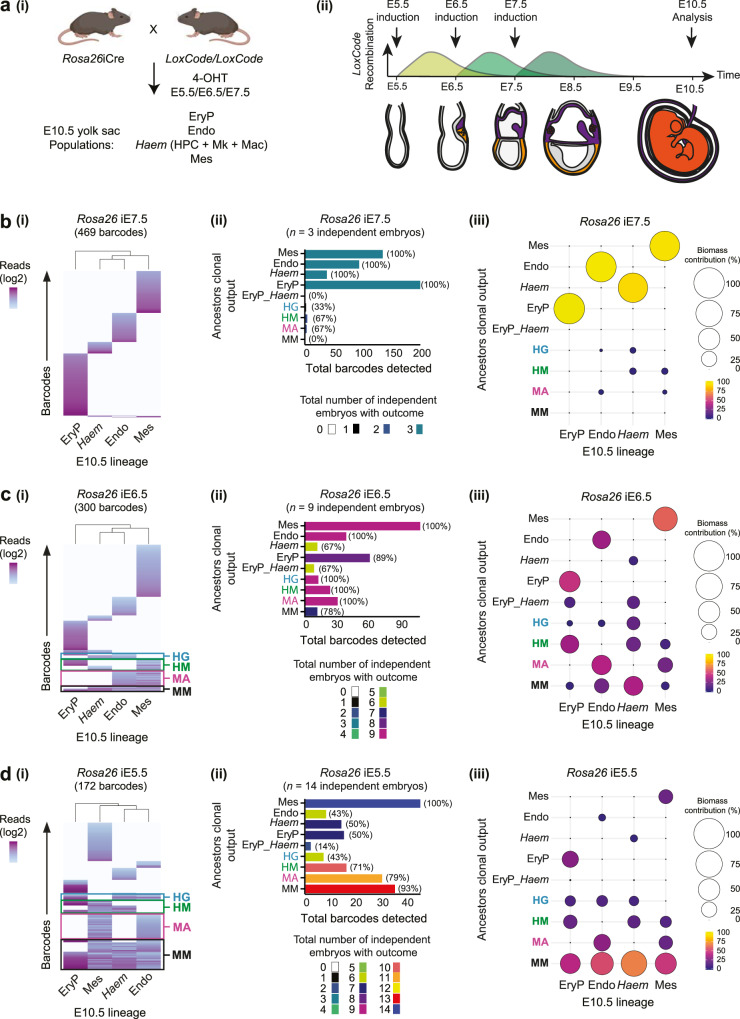
Identification of the in vivo haemangioblast and discovery of the haematomesoblast. **a** (i) Experimental design to assess lineage relationships between all mesoderm derivatives of the yolk sac. Created with BioRender.com. (ii) Barcoding window in reference to 4-OHT injection time. Experimental mice received only one dose of 4-OHT (at either E5.5, E6.5, or E7.5). **b** Induction of barcode formation at E7.5 induction using *Cdh5*iCre (469 barcodes, *n* = 3 embryos). (i) Heatmaps of all informative barcodes. (ii) Biological reproducibility of clonal outcomes. Colours represent the total number of independent embryos with the stated clonal outcome. Values in parentheses represent the percentage of independent embryos in which the clonal outcome was observed. (iii) Summary of contribution to the biomass of E10.5 yolk sac lineages (based on 5–9 element barcodes [464 barcodes]). **c** Induction of barcode formation at E6.5 using *Rosa26*iCre (300 barcodes, *n* = 9 embryos). (i) Heatmaps of all informative barcodes. (ii) Biological reproducibility of clonal outcomes. Colours represent the total number of independent embryos with the stated clonal outcome. Values in parentheses represent the percentage of independent embryos in which the clonal outcome was observed. (iii) Summary of contribution to the biomass of E10.5 yolk sac lineages (based on 5–9 element barcodes (based on 5–9 element barcodes [280 barcodes]). **d** Induction of barcode formation at E5.5 using *Rosa26*iCre (172 barcodes, *n* = 14 embryos). (i) Heatmaps of all informative barcodes. (ii) Biological reproducibility of clonal outcomes. Colours represent the total number of independent embryos with the stated clonal outcome. Values in parentheses represent the percentage of independent embryos in which the clonal outcome was observed. (iii) Summary of contribution to the biomass of E10.5 yolk sac lineages (based on 5–9 element barcodes (based on 5–9 element barcodes [163 barcodes]). Mes (mesenchyme), Endo (endothelium), EryP (primitive erythroid), *Haem* (HPC, megakaryocyte, and macrophage), HG (haemangioblast), HM (haematomesoblast), MA (mesenchymoangioblast), and MM (multi-outcome mesoderm).

To investigate the ancestral relationship between haematopoietic, endothelial, and mesenchymal lineages in the yolk sac, we collected E10.5 endothelium, primitive erythrocyte, mesenchyme, and the *Haem* group (Fig. 6a). When *LoxCode*:*Rosa26*iCre mice were induced at E7.5 few barcodes were shared between the mesodermal derivatives (Fig. 6b and Supplementary Fig. 10a) indicating that mesodermal derivatives in the yolk sac were on separate lineage trajectories from E7.5.

Induction of barcode formation at E6.5 yielded a striking pattern of barcode sharing that was consistent with labelling a dynamic developmental continuum that spanned mesoderm with multi-lineage contribution and lineage-restricted trajectories (Fig. 6c and Supplementary Fig. 10b). The E6.5 clonal outcomes revealed a clear picture of how the mesoderm diversifies into its four major outcomes, this included:

1. Multi-outcome mesoderm that contributed to mesenchyme, conventional endothelium, primitive erythrocytes, and/or *Haem* lineages (i.e., haematogenic endothelium).
2. Haemangioblasts that were restricted to the formation of conventional endothelium and primitive erythrocyte/*Haem* outcomes. Thus were capable of producing both haematogenic endothelium and conventional endothelium.
3. Mesenchymoangioblasts^48^ that were restricted to the production of conventional endothelium and mesenchyme.
4. A previously undescribed class of precursor that we have termed the haematomesoblast. The haematomesoblast outcome was restricted to mesenchyme and primitive erythrocyte/*Haem* lineages. Thus, were capable of producing haematogenic endothelium but not conventional endothelium.

Each class of dual-outcome precursor was observed in all of the nine embryos induced at E6.5 (Fig. 6cii), which demonstrates the biological reproducibility and robustness of the observations.

Induction of barcode formation at E5.5 identified haemangioblast, mesenchymoangioblast, and haematomesoblast outcomes as well as a greater number of multi-lineage clones (Fig. 6d and Supplementary Fig. 10c). As recombination occurs for at least 24 h in this system, we cannot pinpoint the exact time of emergence of each type of ancestors, however, our data clearly demonstrated a progression from multi > dual > uni-lineage outcome between E5.5 and E7.5.

## Discussion

Using scRNA-Seq gene expression, we made the surprise discovery that mesenchyme-associated genes were co-expressed with haematopoietic and endothelial genes in the E7.25–E7.75 *Flk1*+ extraembryonic mesoderm. This indicated that a mesenchymal axis of early haematopoietic and endothelial development existed. Using the *LoxCode* mouse to induce cellular barcoding during unperturbed embryonic development, we were able to test our hypothesis in vivo. This powerful approach provided evidence that bona fide haemangioblasts exist in vivo, demonstrated the in vivo relevance of the mesenchymoangioblasts previously identified in vitro^48^, and enabled the discovery of a previously undescribed class of haematogenic precursor, the haematomesoblast which connects the mesenchymal derivatives of the mesoderm to the haematopoietic lineages.

It could be possible that the patterns of dual lineage outcomes observed arose because of lineage bias rather than lack of tripotentiality. Our observed limit of *LoxCode* barcode detection was 1 in 16,000 cells, which provided a 2–5 fold coverage of the non-erythroid yolk sac lineages. Thus, if a precursor such as the haematomesoblast contributed to a third lineage (e.g., the endothelium), the magnitude of this contribution would have been vanishingly small. Additionally, when investigated at E7.5, more than 90 % of the biomass of E10.5 yolk sac macrophages and cephalic microglia was shared (Fig. 3d). This suggested that any possible dropout effect (i.e., causing dual—rather than tri-lineage outcomes) was unlikely to be an issue by virtue of the increased time for clonal amplification (4–5 days compared to 3 days). Indeed, >90% connections were observed for the *Haem* lineages with an E6.5 induction (Fig. 4a) and the percentage of shared barcodes across PCR technical replicates ranged between 96.2 and 99.6% in the E5.5 induction experiments (Supplementary Fig. 10). A caveat of our study is that all end-point analysis was performed at E10.5, therefore it remains possible that clonal outcomes that we observed as dual outcome could give rise to a third lineage later in embryogenesis (e.g., given more time, mesenchymoangioblasts could generate hemogenic endothelium and so contribute to haematopoiesis).

Whether the haemangioblast, mesenchymoangioblast, and haematomesoblast precursors represent stable and isolatable populations with only dual lineage outcomes is unclear. A previous study showed that colonies with endothelial and haematopoietic output also contained mesenchymal derivatives^10^. Although this could be an outcome of the complex culture system required to investigate the differentiation potential of these cells, this could also indicate that all cells with a dual lineage output in vivo are fundamentally tripotential ancestors that only differentiate along two lineage trajectories due to local environmental cues. Heterotopic transplantations have shown that transplanted epiblast cells adopt the fate of their new location rather than that of their region of origin^49,50^. This highlighted the importance of regional cues in lineage differentiation. In the yolk sac,  haematopoietic outcome is largely restricted to the blood band (Fig. 1aii and ref. 24). This could either be due to inhibition of the haematopoietic potential of a tripotent mesodermal ancestor at the level of the endothelial zone or the activity of a specific mesenchymoangioblast precursor. Of note, the finding that from E6.5 blood (particularly EryP) and endothelial lineages are seeded by largely ancestrally distinct clones is in keeping with previous in situ^13^ and ex vivo^14^ tracking studies.

Although the molecular nature of the intermediates remains unclear, our findings indicate that haematopoietic and endothelial lineages are generated via both clonally related and unrelated ancestries.

The existence of two haematogenic precursors that are broadly equivalent in their ability to produce haematopoietic lineages suggests that there are multiple cellular pathways to blood production in the yolk sac, one endothelial affiliated (haemangioblast) and one mesenchymal affiliated (haematomesoblast). The unequivocal role of transcriptional master regulators such as GATA1, GATA2, RUNX1, and TAL1 for in vivo blood cell emergence^8,19,21,32,33,51–63^, and that E10.5 differentiated lineages cluster homogeneously, is consistent with the notion that haemangioblast and haematomesoblast differentiation routes must converge on the same molecular machinery to induce haematopoietic commitment. This likely occurs at the level of the E7.25–E7.75 *Cdh5*^+^ haemato-endothelial precursors, which might have the capacity for *Haem* group or endothelial lineage production but do not generally contribute to both outcomes. Molecular mechanisms describing how this fate sorting could occur in vivo, involving interplay between SOX7, FOXF1, and RUNX1, have been proposed using in vitro embryonic stem cell differentiation models^64–68^.

Regarding the relationship between the haematopoietic lineages in the yolk sac, we have demonstrated that despite previous interpretations of yolk sac megakaryocytes being a primitive lineage co-emerging with the primitive erythrocytes^3,4^, these two lineages diverge between E6.5 and E7.5, prior to the emergence of the haemogenic endothelium. Furthermore, we found that megakaryocyte, macrophage and HPC lineages predominantly derive from a common haematopoietic precursor which yields progeny that diverge between E7.5 and E8.5 and continue to develop in parallel in the yolk sac without substantial trajectory cross over before E10.5.

**Fig. 7:**
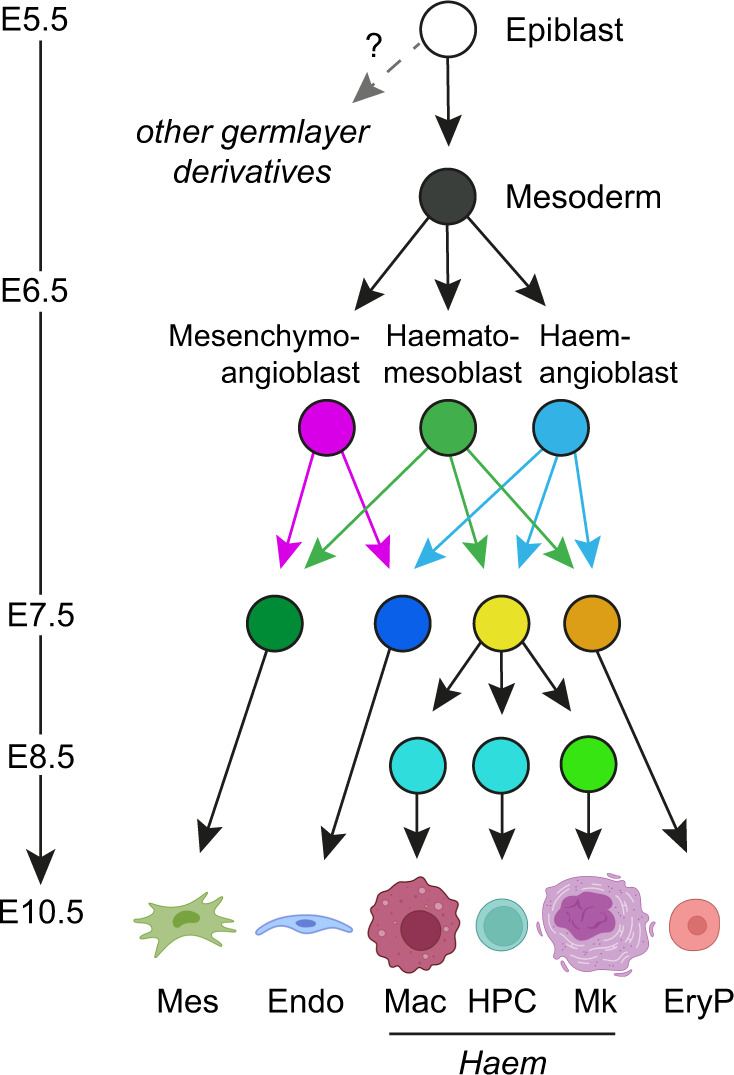
Model of mesodermal diversification in the yolk sac via dual outcome precursors. Endothelial, mesenchymal, and haematopoietic lineages in the yolk sac are the product of mesenchymoangioblast, haemangioblast, or haematomesoblast differentiation. Created with BioRender.com. EryP (primitive erythroid), Mk (megakaryocyte), HPC (haematopoietic progenitor/colony forming cell), Mac (macrophage), *Haem* group (Mk+ HPC+Mac), Endo (endothelium), Mes (mesenchyme).

In summary, these data demonstrate the in vivo existence of the haemangioblast, the in vivo activity of mesenchymoangioblasts, and the discovery of a new class of haematogenic precursor—the haematomesoblast. The haemato-endothelial lineages of the yolk sac are established by the output of this precursor trio (Fig. 7).

## Methods

### Mice

*Flk1*-gfp^69^, *Cdh5*ERT2Cre^44^, *Rosa26*ERT2Cre^70^, and *Rosa26R*eYFP^71^ lines were maintained on a C57BL/6 background. All animal experiments were approved by The Walter and Eliza Hall Institute animal ethics committee.

### Confocal imaging

Embryos were fixed in 2% PFA for 20 min at room temperature. Samples were blocked and permeabilized in 0.6% Triton-X/10 % FCS/Ca^2+^/Mg^2+^ DPBS for 30 min at room temperature. Staining with primary and secondary antibodies (Supplementary Data 1) was performed either overnight at 4 °C or for 6–8 h at room temperature in 0.6% Triton-X/10% FCS/Ca^2+^/Mg^2+^ DPBS. Nuclei were stained with DAPI for one hour at room temperature. Embryos were transferred to 4 ml silanized glass vials (Supelco) and dehydrated in a gradient of tetrahydrofuran (THF, Sigma) in H_2_O (50%, 70%, 100%) with 1.5 h washes held at room temperature, and a final overnight incubation in 100% THF held at 4 °C^72^. The next morning embryos were transferred to a coverslip mounted with a silicone Fastwell (Grace BioLabs) and cleared in two changes of 100% dibenzyl ether (DBE, Sigma) before imaging on a Zeiss LSM780 confocal microscope. Data were processed using Imaris Software (v9, Bitplane).

### Flow cytometry

E7 yolk sacs were dissected and dissociated in 0.25% Trypsin/EDTA (Gibco) at 37 °C for five minutes^73^. Samples were washed with 1 ml of FACS buffer (7% FCS/ Ca^2+^/Mg^2+^-free DPBS) and centrifuged at 500 × *g* for 5 min. Samples were resuspended in 1 ml of FACS buffer and mechanically disrupted by gentle trituration with a P1000 20 times, then filtered through a 40 μm nylon mesh filter and centrifuged again before being placed on ice. Tissues from all older developmental stages (E8–10.5 yolk sacs) were dissociated in 10% Collagenase-Dispase solution (5 mg/ml stock; Roche) made in Dissection Medium (7% FCS/ Ca^2+^/Mg^2+^ DPBS) at 37 °C for 45–60 min, washed and mechanically disrupted as described above. Single cell suspensions were maintained on ice. Cells were washed and stained in FACS buffer. Staining of single cell suspension was performed with primary antibodies for 1 h. Dead cells were excluded according to uptake of 7-aminoactinomycin D (7-AAD). Gate placement was determined using appropriate isotype and fluorescence minus one controls. Cells were analysed on either BD LSRFortessa or LSRII cytometers. Flow cytometry cell sorting was performed on BD FACSAria using a 100 μm nozzle with collection in 1.5 ml eppendorf tubes containing 700 μl FACS Wash to minimise cell loss with collection tubes and cells maintained at 4 °C throughout the sorting process. Sorted cells were always reanalysed to determine sort purity. Data were analysed using FlowJo software.

#### Fluidigm C1 single cell dataset

##### Capture and cDNA generation

Populations of interest were individually purified by flow cytometry sorting (Supplementary Data 1). Cell counts were performed by haemocytometer; 6000 cells were prepared for processing according to the manufacturer’s instructions for capture on the Fluidigm C1 integrated fluidic circuit (IFC) with the capacity for 96 individual cells and a 10–17 μm capture aperture. All scripts used were for the 10–17 μm IFC. Briefly, Solutions A–C were prepared and held on ice, then the IFC was primed before 3000 cells were loaded in 20 μl of 3: 2 FACS Wash: C1 Suspension Reagent for capture (150 cells/μl). After inspecting and imaging each capture site to record the presence and quantity of captured cells and their morphology, Solutions A–C were loaded into the IFC according to the Loading Map, and overnight cDNA and pre-amplification was performed. The following morning ~3 μl of cDNA was harvested into 10 μl of C1 DNA Dilution Reagent in a 96 well plate. Four single cell samples were run on a Tapestation as quality control to assess whether the overnight cDNA step was successful, before storing the plate at −20 °C until sequencing library preparation.

##### Single-cell RNA library preparation and sequencing

cDNA concentration was assessed for each cell sample using the Qubit or a PicoGreen plate reader as per the manufacturer’s instructions. Libraries were prepared using the Nextera XT kit and Illumina 96 index kits according to the Fluidigm protocol modified from the Illumina protocol to use ¼ of the kit per 96 well plate. Briefly, cDNA concentrations were adjusted to be 0.1–0.3 nm/μl using C1 Harvest Reagent. Tagmentation adapters were added to the cDNA in the process of amplification, then Illumina sequencing primers were adapted along with P5 and P7 Single Cell Indices during low cycle number (×12 cycle), full-length amplification PCR. 50–90 single cells were pooled to sequence on a High-Seq lane depending on the single cell capture efficiency for each population, with sample quality control performed on the Tape Station after AMPure XP (Agencourt) magnetic bead clean-up to confirm fragment size enrichment (200–1000 bp). RNA Sequencing was performed using Illumina HiSeq with Paired-End 100 bp reads, and a pool of up to 96 cells occupied each lane.

##### Bioinformatics analysis of single cell Fluidigm data

Fastq files were aligned to Ensembl mouse genome version 84 using Rsubread package (doi:10.18129/B9.bioc.Rsubread). The featureCounts function from the same package was used to generate counts matrix summarised at the gene level. The data from the 1073 cells captured by Fluidigm technology was filtered using the scater R package (doi:10.18129/B9.bioc.scater) producing a matrix with 926 samples and 15,967 genes. The scran R package (doi:10.18129/B9.bioc.scran) was used to normalise the matrix using computeSumFactors function, and cpm values were generated using calculateCPM function. Multiple dimensionality reduction techniques were applied in order to check that results were robust—including TSNE, PCA, UMAP and more, across a range of parameters. The results shown here were obtained using the python umap package, with n_neighbors=40 and min_dist=0.8. Marker genes for each sorted population (Supplementary Data 3) were obtained using Scanpy’s rank_gene_groups method, using “Wilcoxon” as the method parameter. Heatmaps were generated using Morpheus software (https://software.broadinstitute.org/morpheus/).

##### General statistical analyses (outside scRNA-seq data)

Prism 7 (GraphPad) was used for data analysis and graph production. Data represented as mean ± standard deviation (SD), and analysed using Student’s t-test (two-way, unpaired). One-way ANOVA (using Tukey’s *P* value adjustment) was used for multiple comparisons. Differences were considered statistically significant when *p* < 0.05, designation of ‘ns’ indicates differences were not significant. * = *p* < 0.05, ** = *p* < 0.01, *** = *p* < 0.001, **** = *p* < 0.0001. ‘*n’* was used to designate the number of independent experiments.

##### Construction of the *LoxCode* mouse

The *LoxCode* construct was assembled using degenerated oligonucleotides containing a high diversity of element sequences that were sequentially clone into pBlueScriptIISk. Sequencing revealed that all barcode elements differed from at least 2 nucleotides in either orientation. The *LoxCode* mouse was created using CRISPR technology. Cas9-gRNA ribonucleoproteins (guide RNA sequence: 5′-CTCCAGTCTTTCTAGAAGAT-3′) and a circularised vector (pBlueScriptIISk backbone) containing the *LoxCode* cassette were injected into C57BL/6J oocytes before reimplantation into pseudopregnant females. Pups were screened by PCR for presence of the insertion. Sanger sequencing was performed to confirm the *LoxCode* sequence. The *LoxCode* line was bred to homozygosity on a C57BL/6 background. For distribution of *LoxCode* mice, contact corresponding authors.

##### *LoxCode* control experiments

Granulocyte-Macrophage Progenitors from an adult *LoxCode*/*Rosa26*ERT2Cre were transduced with pMSCV-IRES-YFP construct containing the *MLL-AF9* fusion gene (REF: https://www.ncbi.nlm.nih.gov/pmc/articles/PMC2936245/) and transplanted into a Ly5.1 mouse to induce Acute Myeloid Leukaemia (AML). When the leukaemic burden (YFP) in the peripheral blood reached >25%, mice were culled via cervical dislocation and bone marrow cells were harvested and subjected to 1 h of 4-hydroxytamoxifen (4-OHT, Sigma-Aldrich) exposure in vitro (100 nM) followed by three washes. After 48hrs of recovery, single barcoded AML cells were sorted in 96 well plates and expanded. DNA samples were analysed by PCR to assess recombination. Two pools containing various numbers of cells from 11 clones were sorted and analysed: Pool 1 (1, 2, 8, 64, 128, 256, 2564, 4096, 8192, 17456, and 32768 cells) and Pool 2 (1, 4, 8, 128, 256, 512, 3106, 4096, 8272, 16384, and 32768 cells). In two independent 65,535 cell pools, we detected four barcoded cells added per pool but not one or two cells per pool. This means that to detect all barcoded cells in a sample, the barcode frequency must be ≥ 1 in 16,000 cells (Supplementary Data 4). As these experiments were done with purified clones, they were used to set up detection thresholds and removal of potential PCR artefacts (Supplementary Fig. 4c). They were also used to screen barcodes size classes regarding linearity of output (sequenced barcodes) versus input (inputed number of cells) (Fig. 2c).

##### Isolation of barcoded populations

Embryos were generated by crossing *LoxCode*/*LoxCode* mice with *Cdh5*ERT2Cre/+ or *Rosa26*ERT2Cre/*Rosa26*ERT2Cre mice. Noon of the day a vaginal plug was found was counted as E0.5. Barcoding was induced by injection of 4-OHT between E6.5 and E8.5 following a protocol optimised for each line for maximum informative barcode recovery: *Cdh5*ERT2Cre crosses—intraperitoneal injection of 300 μg/mouse of 4-OHT (dissolved in corn oil, Sigma-Aldrich); *Rosa26*ERT2Cre crosses: intravenous injection of 100 μg of 4-OHT (dissolved in KolliPhor, Sigma-Aldrich)^74^. Induced mice were kept in separate cages to prevent untimely induction via tamoxifen shedding. Yolk sac or head cell populations were recovered at E10.5. Concepti were dissected out of the uterus in (37 °C 7% Fetal Calf serum, DPBS with Ca^2+^ and Mg^2+^) and rinsed three times in this buffer. The umbilical cord was pinched and cut beneath the placenta and the embryo and yolk sac transferred to a clean dish of (37 °C 7% Fetal Calf serum, Ca^2+^/Mg^2+^-free DPBS, FACS buffer). The yolk sac was dissected with scissors (avoiding pulling on the tissue to preserve endothelial cells) and the embryo quickly moved to a fresh plate. The yolk sac and blood spilled from the umbilical cord were collected. Embryos were screened for normal development and heartbeat, staged by general morphology and somites counts and used for genotyping before sorting. For head macrophage (microglia) purification, heads were dissected after embryo scoring. Yolk sac and head samples were rinsed in DPBS and dissociated enzymatically with Liberase (100 μg/ml in Ca^2+^/Mg^2+^-free DPBS) for 12 min at 37 °C. The reaction was stopped by adding 2 ml of cold FACS buffer and immediate centrifugation. Samples were resuspended in 1 ml of FACS buffer with 2.5 mM EDTA, incubated for a few minutes on ice to weaken cell adhesion further, and mechanically dissociated with a P1000 pipetman. Samples were filtered through a 40 μM nylon mesh, centrifuged, and resuspended for antibody labelling. Antibodies were purchased from Biolegend (PDGFRA (APA5), PECAM (390), CX3CR1 (SA011F11)), Invitrogen (CD41 (eBioMWReg30)) or made in-house (Ter119) and CD45 (30-F11). After antibody staining (1 h, 4 °C), cells were washed and counterstained with 7AAD (Invitrogen) for dead cell exclusion. Cells were sorted on an Aria Cell Sorter (Becton Dickinson) and collected in FACS buffer. An aliquot of cells was used to assess sample purity with a 95% threshold (Supplementary Data 5), and the remainder was immediately centrifuged after sort. Cell pellets were lysed with 100 μg/ml Proteinase K in proteinase K buffer, digested for 2 h at 56 °C, inactivated for 1 h at 85 °C and 5 min at 95 °C. Lysates were maintained at −20 °C until library preparation.

##### *LoxCode* library preparation

*LoxCode* DNA was amplified by PCR using primers complementary to *LoxCode* flanking arms (5′-TCTAGAGGATCCCCGGGTACCGA−3′ and 5′-TGATCCGCGCCTGGATGAAT−3′) with the following programme (98 °C 2 min, 22× (98 °C 20 s, 65 °C 20 s, 72 °C 30 s)). Illumina sequencing primers, a stagger to increase library diversity, indexes (96 indexes/sequencing lane), and P5/P7 flow cell adapters were introduced in 2 subsequent 5-cycle PCR steps (98 °C 2 min, 5× (98 °C 20 s, 70 °C 20 s, 72 °C 30 s)), designed following Illumina guidelines. Additional 5-cycles of amplification were performed using phosphorothioated P5 (5′-A*A*T*GATACGGCGACCACCGAGATCTA*C*A*C-3′) and P7 (5′-C*A*A*GCAGAAGACGGCATACGA*G*A*T-3′) primers to ensure long term storage. PCR Primers were removed and barcodes with more than one element size-selected after each step using NGS Magnetic-bead clean up (Mackery-Nagel). Libraries were quantified on a Tapestation (Agilent), pooled, and sequenced using Illumina MiSeq kits (600 cycles).

##### Analysis of the *LoxCode* sequencing data

All analyses were carried out using custom C++ and R scripts (available on request). Raw paired-end dual-indexed sequencing data was demultiplexed into individual samples. For each sequence, *LoxCode* elements (stereotypical in position) were extracted and aligned to those of the original cassette. To compute the minimal number of recombination steps necessary to make each *LoxCode* (complexity), a reference table with all possible combinations was created. For this, a simulation of all possible recombinations (excisions and inversions) of the original *LoxCode* construct was carried out, assuming a 82 bp minimal distance between loxP sites^75^. The resulting barcodes were stored and attributed a complexity of one. In a second step, all entries of this table were subjected to the same process. Barcodes generated in that way already present in the table were discarded, while new barcodes were added and attributed a complexity of two. This process was repeated 15 times until no new barcodes were generated, establishing the minimum number of recombination events needed to create any specific barcode and an expected theoretical diversity of 30,204,722,030 barcodes^38^. The usage of paired-end MiSeq2 600 cycles kits only allowed the sequencing of 12 out of 13 elements. *LoxCodes* with less than 13 elements (the vast majority of barcodes) were sequenced in full with this protocol. For 13-element *LoxCodes*, the sequence and orientation of the middle element was inputed from its surrounding elements, assuming a minimal number of recombination steps.

To exclude barcodes that could be illegitimate or made independently in two cells of the same embryo, barcoding data was processed following the flowchart on Supplementary Fig. 5: barcodes not conforming to the expected structure or with limited diversity (1-element, 13-element unrecombined barcodes) were removed. Barcodes potentially resulting from PCR artefacts (Supplementary Fig. 4c) were filtered out if their reads represented less than 10% of those of all their potential parents combined (ie any barcode containing all the elements of the potential offspring barcode). A detection threshold of 100 reads was used to remove sequencing errors and potential contamination. Illegitimate barcode filtering and detection thresholds were determined using control experiments described above. Remaining (legitimate) barcodes were normalised for reads per cell across each dataset. As some barcodes were detected in all or many embryos, this raised the possibility of repeat barcode generation in independent cells. We found that barcode classes defined by length and complexity had various inherent combinatorial diversity (Fig S5 Box 1) and that high diversity (>2000 possible combinations) classes had a higher chance of being unique to one embryo in our first two datasets (11 embryos) (Supplementary Fig. 5 Box 2). We used this information to filter for barcodes with the highest probability of clonality: belonging to length/complexity classes with the highest diversity and likelyhood to be detected in one embryo only (coloured orange in Supplementary Fig. 5) and uniquely detected in each dataset analysed. Barcodes passing all filtering steps were termed informative barcodes. Heatmaps were generated using Heatmap.2 (gplots R package). Barcode behaviour (display of number of barcodes with a given behaviour per experiment) and biomass (% of cells of a given lineage deriving from ancestors with a particular fate) analyses were generated using custom R scripts. For biomass analysis, only 5–9 elements barcodes were included, as 1–3 and 13 elements barcode frequencies were affected by beads clean-up, PCR, and sequencing biases. Total numbers of barcodes generated in each experiment as shown in Supplementary Data 8.

##### Duration of barcode formation after 4-OHT administration

Embryos were generated by crossing *LoxCode*/*LoxCode* or *LoxCode*/*+* mice with *Rosa26*ERT2Cre/*Rosa26*ERT2Cre mice. Noon of the day, a vaginal plug was found was counted as E0.5. Barcoding was induced by injection of 100 μg of 4-OHT (dissolved in KolliPhor, Sigma-Aldrich) at E6.5 by intravenous injection. Whole concepti were collected 1, 6, 12, and 24 h after induction, and yolk sacs only after 48 and 120 h. 8–14 embryos were collected for each timepoint. Samples were processed as described above and sequenced on MiSeq using a 600 cycles kit. Barcode sequences and complexity were extracted as previously described. For each embryo, the proportion of reads or barcodes dedicated to quantifiable (complex) barcodes was determined.

##### 10× Genomics scRNA-Seq dataset

Populations of interest were individually purified from a pool of 33 C57BL/6 E10.5 embryos by flow cytometry (Supplementary Data 1), as described on Supplementary Fig. 4d. To enable the identification of the population of origin, each population was labelled with a distinct MultiSeq hashtag (^76^). 17,000 cells were loaded on the 10× Genomics Chromium system. 13,780 cells were identified using CellRanger. A high quality 7316 cells dataset was obtained after screening for transcriptome quality (>3000 UMI, >1000 features, <5% mitochondrial transcripts) and excluding cells with inconclusive hashtag calls or multiplets. Transcriptomes were scaled using ScTransform. Seurat clusters were identified and annotated using DE gene lists. Good correlation was found between hashtag call and transcriptional identity. In particular, >97% of cells purified as Mes belonged to the mesenchymal cluster (Fig. 5a–c). Others were most likely sorter errors or uncalled doublets. Cells belonging to the mesenchymal cluster were re-scaled and clustered for further identification. Aside from endothelial, haematopoietic, and mesenchymal populations, a small number of extraembryonic endodermal cells were identified (19 cells, cluster 22, Fig. 5a). The majority of those cells were labelled with an endothelial hashtag, most likely due to autofluorescence in the BV421 channel (CD31). Those cells, known to diverge from the epiblast lineage at E4.5^77,78^, represent a minimal (<4%) contamination of the endothelium, with no ancestral relationship to any of the followed populations in the frame of these experiments, and would therefore appear as endothelium only-barcodes in these experiments.

### Reporting summary

Further information on research design is available in the Nature Portfolio Reporting Summary linked to this article.

## Supplementary information

Supplementary Information

Description of Additional Supplementary Files

Supplementary Data 1

Supplementary Data 2

Supplementary Data 3

Supplementary Data 4

Supplementary Data 5

Supplementary Data 6

Supplementary Data 7

Supplementary Data 8

Supplementary Software 1

Supplementary Software 2

Reporting Summary

## Data Availability

The Fluidigm scRNA-Seq dataset of E7.25 - E10.5 yolk sac lineages have been deposited in NCBI’s Gene Expression Omnibus under accession code GSE164336. The 10X scRNA-Seq dataset of E10.5 yolk sac lineages have been deposited in NCBI’s Gene Expression Omnibus under accession code GSE204896. Differential expression analysis of scRNA-Seq has been provided in Supplementary Data 3, 6, and 7. *LoxCode* mice and/or raw or processed data presented in this manuscript will be made available on request.

## Code availability

All codes used will be made available upon request.
