# Supplementary material for: In vivo clonal tracking reveals evidence of haemangioblast and haematomesoblast contribution to yolk sac haematopoiesis
Source: Nat Commun. 2023 Jan 3;14:41. doi: 10.1038/s41467-022-35744-x (PMC9810727; doi:10.1038/s41467-022-35744-x)
Supplement: Supplementary file 1 — Supplementary Information [file 41467_2022_35744_MOESM1_ESM.pdf]

Biben *et al* Supplementary Information

Supplementary Software captions

Supplementary Software 1. Interactive 3D UMAP of single-cell RNA-Seq data with cells coloured according to lineage immunophenotype (html file that can viewed in a web browser).

Supplementary Software 2. Interactive hybrid 3D UMAP of single-cell RNA-Seq data coloured according to lineage immunophenotype or transcriptional cluster (html file that can viewed in a web browser).

Supplementary Figures

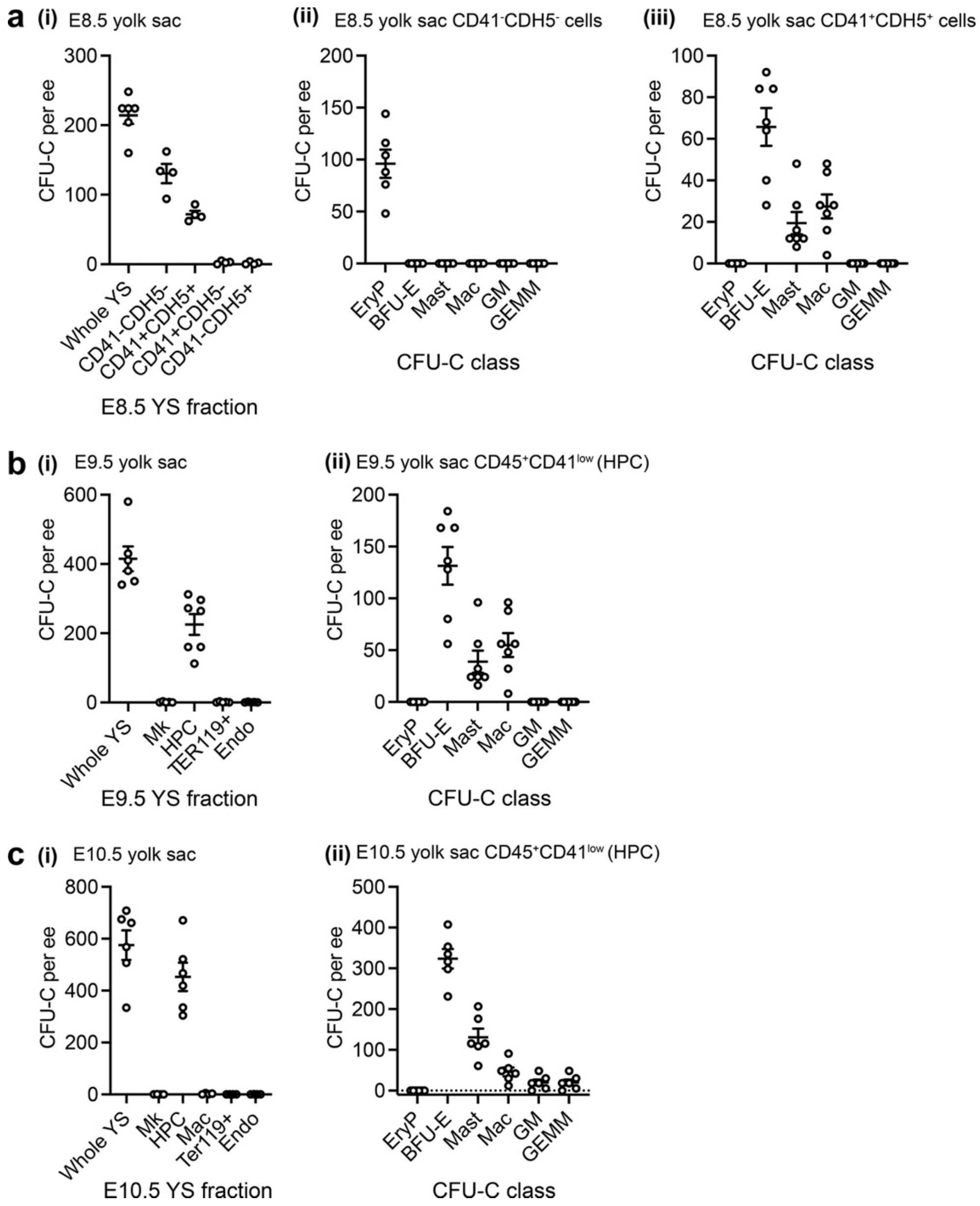

Supplementary Fig. 1: Immunophenotypic distribution of Colony Forming Unit - Cell (CFU-C) activity in E8.5 – E10.5 yolk sacs.

a, (i) CFU-C distribution in one embryo equivalent (ee) of E8.5 yolk sac (YS) from whole or FACS purified populations. Distribution of CFU-C class in CD41-CDH5- (ii) and CD41+CDH5+ (iii) cells from E8.5 YS. Experiments were performed on pooled embryos (between 4 – 8 embryos per pool), *n* = 4 – 6 independent pools performed over 4 – 6 experimental days. Bars represent mean ± SEM.

b, (i) CFU-C distribution in one embryo equivalent (ee) of E9.5 yolk sac (YS) from whole or FACS purified populations. Distribution of CFU-C class in CD45+CD41low (ii) cells from E9.5 YS. Experiments were performed on pooled embryos (between 4 – 10 embryos per pool), *n* = 4 – 7 independent pools performed over 4 – 6 experimental days. Bars represent mean ± SEM.

c, (i) CFU-C distribution in one embryo equivalent (ee) of E10.5 yolk sac (YS) from whole or FACS purified populations. Distribution of CFU-C class in CD45+CD41low (ii) cells from E10.5 YS. Experiments were performed on pooled embryos (between 4 – 8 embryos per pool), *n* = 4 – 6 independent pools performed over 4 – 6 experimental days. Bars represent mean ± SEM.

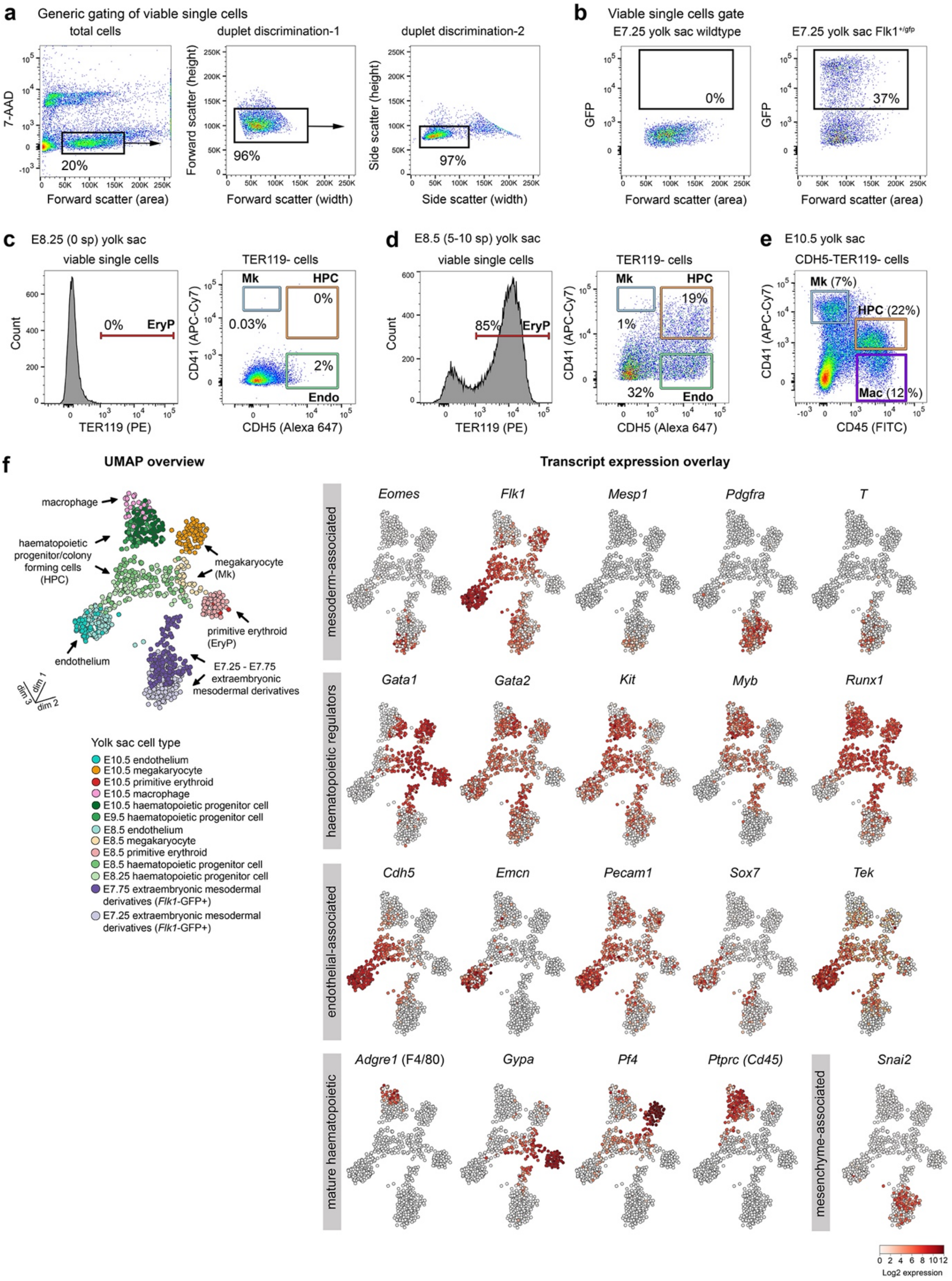

Supplementary Fig. 2: Flow cytometry gating strategies used to identify lineages used for scRNA-Seq expression profiling.

a, Gating strategy used to select for viable (7-AAD-) single cells for flow cytometric analysis and sorting.

b, Gating strategy used to identify *Flk1*-GFP+ cells from viable single cells suspensions of E7.25 and E7.75 yolk sac. Representative examples shown are from E7.25 yolk sacs (*n* = 12 independent experiments, comprised of yolk sacs from 5 – 15 embryos per experiment).

c – e, The E8.5 yolk sac contains three identifiable haematopoietic lineages (EryP, Mk, and HPC) [1] and the endothelium (Endo). These lineages can be identified according to the differential expression of TER119, CDH5 (also known as VE-CADHERIN), and CD41 [1-4]. Although Mac-associated genes can be detected at E8.5 [5], the lineage is not prominent until E10.5. By E10.5, haematopoietic lineages are resolved using a combination of TER119, CD41, and CD45 expression. Thus, between E7 – E10.5, five discrete haematopoietic or endothelial lineages emerge (Endo, EryP, Mk, HPC, and Mac). Shown in the gating strategy used to identify EryP, Endo, Mk, Mac, and HPC lineages from the yolk sac at E8.25 **(c**, *n* = 12**)**, E8.5 **(d**, *n* = 11**)**, and E10.5 **(e**, *n* = 9**)**. Examples shown derive from the analysis of pooled yolk sac (7 – 20 tissues per pool).

f, Expression of key lineage-associated gene expression overlaid on UMAP plot of scRNA-Seq data. Examples shown are of mesoderm-associated genes (*Eomes*, *Flk1*, *Mesp1*, *Pdgfra*, and *T*), key haematopoietic transcription factors (*Gata1*, *Gata2*, *Kit*, *Myb*, and *Runx1*), endothelial-associated genes (*Cdh5*, *Emcn*, *Pecam1*, *Sox7*, and *Tek*), mature haematopoietic lineage genes (*Adgre1*, *Gypa*, *Pf4*, and *Ptprc*), and the mesenchyme-associated gene *Snai2*. The observed patterns of gene expression bench mark the quality of the dataset.

Endo (endothelium), EryP (primitive erythroid), Mk (megakaryocyte), HPC (haematopoietic progenitor/colony forming cell), Mac (macrophage).

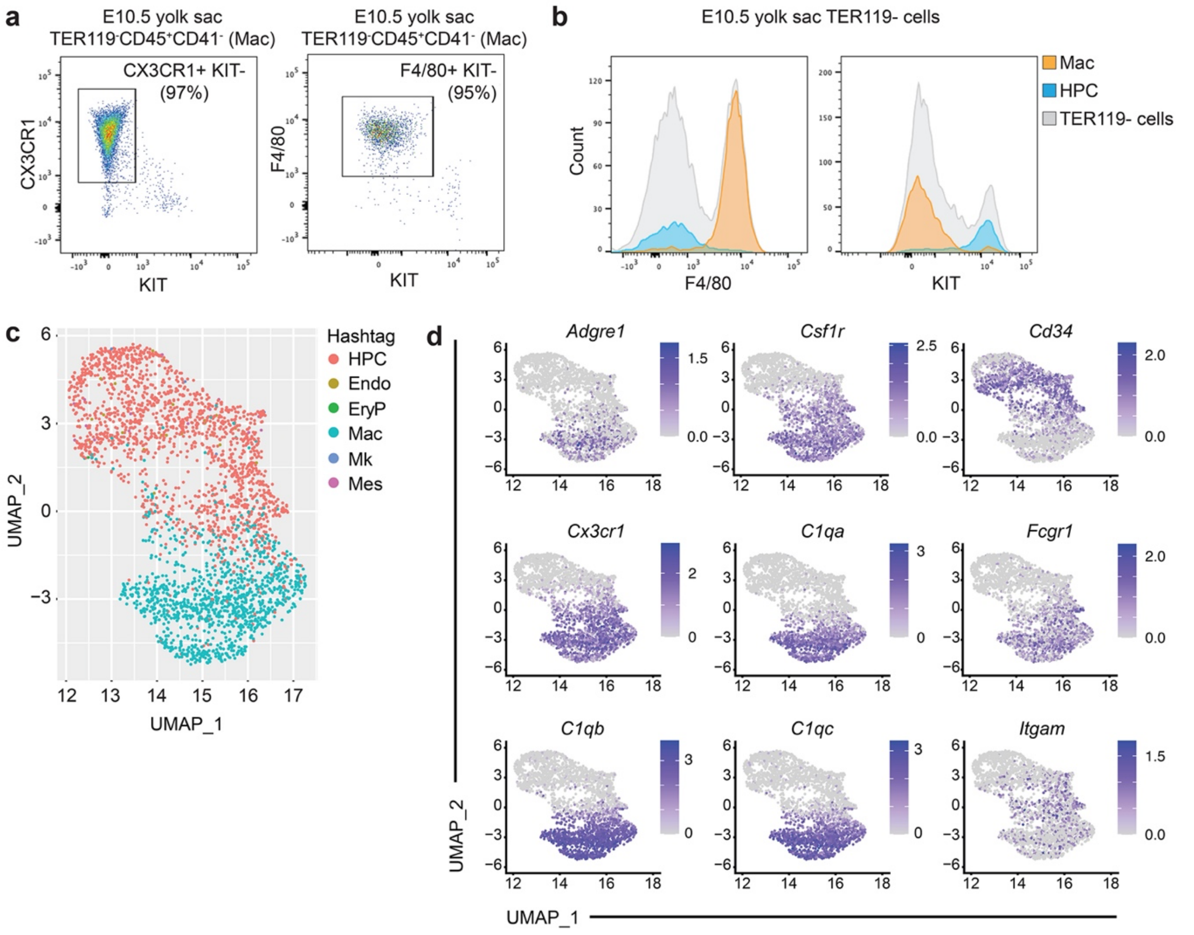

Supplementary Fig. 3: E10.5 yolk sac HPC and macrophage populations.

a, CX3CR1 and F4/80 detection by flow cytometry on the E10.5 yolk sac Mac (TER119- CD45+ CD41-) population.

b, F4/80 and KIT detection by flow cytometry on E10.5 yolk sac Mac (TER119- CD45+ CD41-) and HPC (TER119- CD45+ CD41+) populations.

c and d, Single cell RNAseq (10X, see Figure 5) on E10.5 yolk sac HPC and Mac populations: **(c)** Hashtag (population of origin) of HPC and Mac populations, note that very small numbers of cells with Endo, EryP, Mes, and Mk hastags are found intermixed with the Macs and HPCs.

(d) Macrophage differentiation markers expression in HPC and Mac subsets.

Endo (endothelium), EryP (primitive erythroid), Mk (megakaryocyte), HPC (haematopoietic progenitor/colony forming cell), Mac (macrophage), Mes (Mesenchyme).

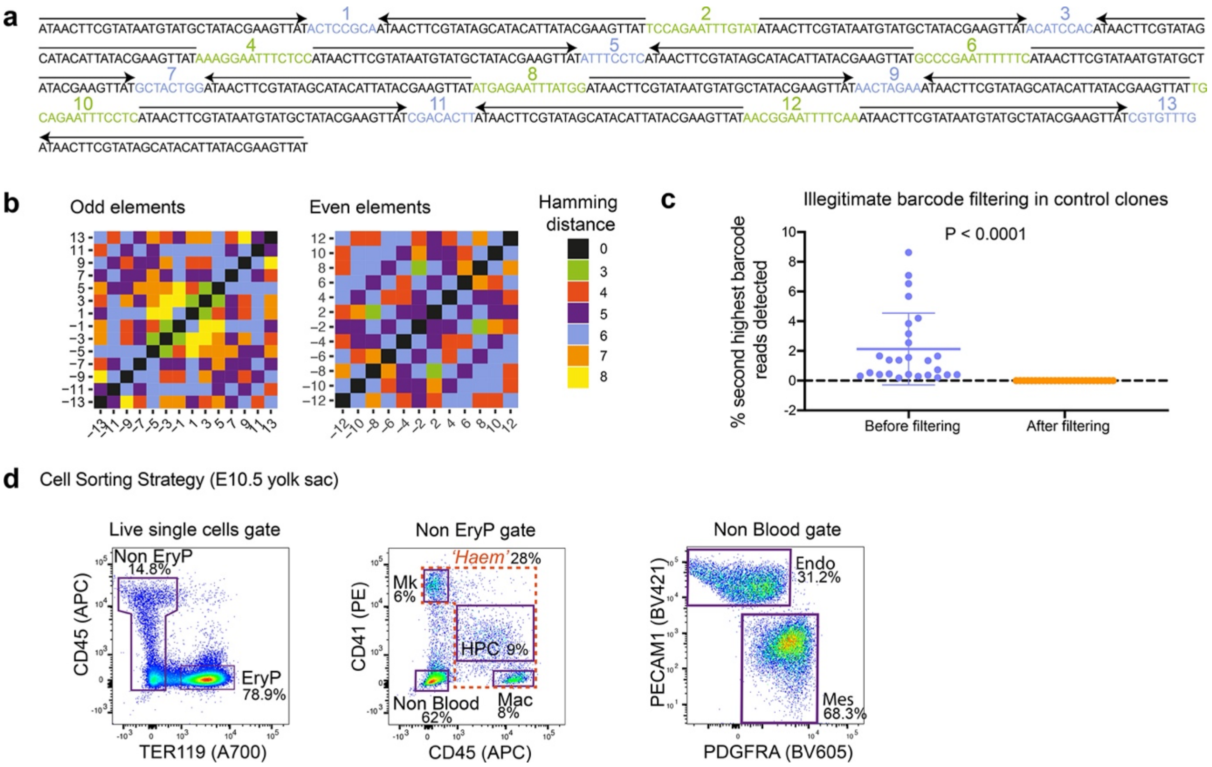

Supplementary Fig. 4: Sequence and technical testing of the *LoxCode* construct.

a, *LoxCode* construct sequence. LoxP sites (arrows). Odd (8bp) and even (14bp) elements are indicated.

b, Hamming distances (number of nucleotide differences) between sequences of odd or even elements in either orientation. All elements have a Hamming distance >2 in either direction ensuring robust identification following sequencing.

To assess the accuracy, sensitivity and linearity of barcode detection, control experiments were performed with barcoded *LoxCode*/*Rosa26*CreERT2 AML clones. After *in vitro* exposure to 4-OHT, single AML cells were sorted into individual wells and expanded *in vitro* yielding clonal lines that were sequenced for barcode identification (**c**) or pooled in known proportions to assess sensitivity and linearity of barcode detection in a pool (**d**).

c, Proportion of reads from the second more highly detected barcode (the first one being the seeded barcode) in control clones before and after illegitimate barcode filtration. Illegitimate barcodes detected in these samples were derived from the seeded barcode and never exceeded 10% of the "parental" barcode. n = 30 independent barcode libraries. Bars, mean ± SD. Data were analyzed using unpaired two-tailed t-test. Exact p-value is shown.

d, Cell sorting strategy for E10.5 yolk sac mesoderm derivatives.

Endo (endothelium), EryP (primitive erythrocyte), Mk (megakaryocytes), HPC (Haematopoietic Progenitor/Colony forming cell), Mac (macrophage), *Haem* group (Mk + HPC + Mac), Mes (mesenchyme).

1. *LoxCode* locus sequencing:

MiSeq 600 cycles kit

↓

2. *LoxCode* barcode identification:

Alignment of sequence reads to *LoxCode* elements

↓

3. Determination of barcode complexity:

For each barcode sequence, calculation of the minimal number of recombination steps required to generate each barcode

↓

4. Identification of legitimate barcodes:

Removal of illegitimate barcodes (PCR artefacts or sequencing errors, Fig S4C)

↓

5. Normalisation (barcodes counts/cell)

↓

6. Select clonal barcodes:

(a) Only include barcodes with a ***size and complexity leading to a diversity > 2000*** (Box 1, orange cells)
(b) Only include barcodes detected in ***one embryo*** in the dataset.

Most of the barcodes selected in step (a) are uniquely detected in a test dataset (Box 2, *n* = 11 embryos).

Box 1. Values = theoretical diversity for each barcode category

Complexity of barcode	Size of barcode: 3E	5E	7E	9E	13E
1	4	6	8	10	37
2	52	106	162	253	912
3	360	1212	2378	4418	19812
4	1306	11333	31928	70538	317578
5	2006	56242	252490	728021	3988251
6	2016	154973	1403382	5643564	36058157
7	2016	199695	4789612	29519488	238462747
8	2016	201587	9951050	106581710	1149595506
9	2016	201600	12504942	256471281	4056050952
10	2016	201600	12889288	409271561	10401368516

Box 2. Values = % barcodes unique to one embryo for each barcode category. Orange cells are transposed from Box 1.

Complexity of barcode	Size of barcode: 3E	5E	7E	9E	13E
1	0	0	7.7	0	42.9
2	29.5	35.4	72.9	82.1	100
3	74.4	82.1	93.9	99	100
4	90.9	97.9	97.8	96.7	100
5	100	99.5	96.2	100	100
6	100	100	100	100	100
7	n/a	100	100	100	100
8	n/a	100	100	100	n/a
9	n/a	n/a	n/a	100	n/a
10	n/a	n/a	100	n/a	n/a

↓

Informative barcodes used for analysis

Supplementary Fig. 5: *LoxCode* analysis pipeline: identification of informative barcode sequences.

After determining the purity of collected cells (Table S5), *LoxCode* libraries were generated from genomic DNA and sequences were retrieved using Illumina next-generating sequencing. After stringent sequence filtering, barcode reads were normalised per cell. The minimal number of recombination steps required to generate each barcode from the original 13 element cassette was determined (*see Methods* for complexity calculation). Because low complexity barcodes were detectable in most samples, barcode size and complexity were correlated with their likelihood of

detection in independent embryos. To ensure the stringency and reasonable barcode recovery, we imposed the following criteria: only barcodes from reliable barcode classes (orange boxes) and only detected in one embryo per dataset were considered to be informative. Only informative barcodes were used for downstream analyses.

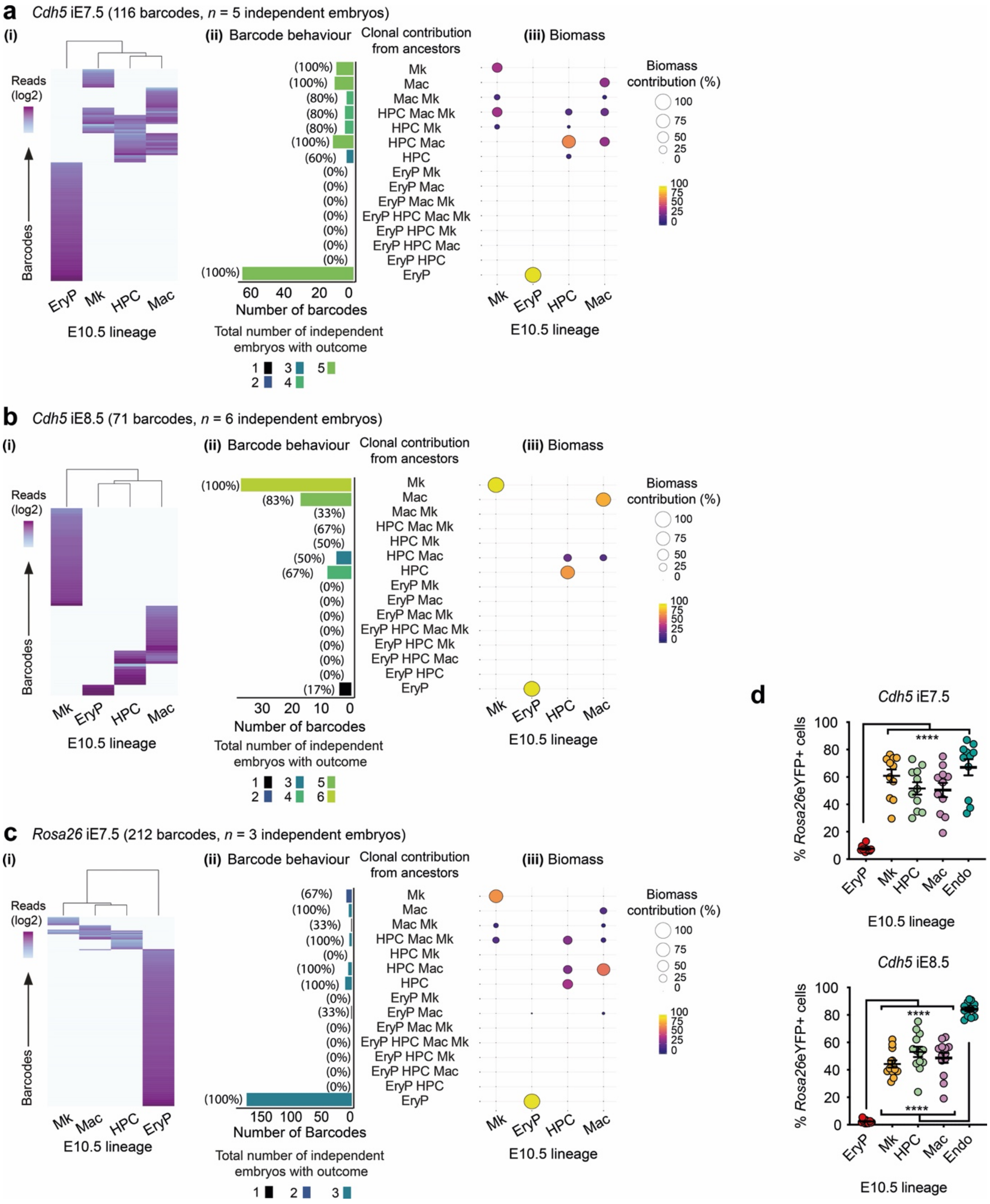

Supplementary Fig. 6: Relationship between EryP and other yolk sac haematopoietic lineages.

a, Induction of barcode formation at E7.5 using *Cdh5*iCre (116 barcodes, *n* = 5 embryos). (i) Heatmaps of all informative barcodes. (ii) Biological reproducibility of clonal outcomes. Colours represent the total number of independent embryos with the stated clonal outcome. Values in parentheses represent the percentage of independent embryos in which the clonal outcome was

observed. (iii) Summary of contribution to the biomass of E10.5 yolk sac haematopoietic lineages biomass (based on 5 – 9 element barcodes [113 barcodes]).

b, Induction of barcode formation at E8.5 using *Cdh5*iCre (71 barcodes, *n* = 6 embryos). (i) Heatmaps of all informative barcodes. (ii) Biological reproducibility of clonal outcomes. Colours represent the total number of independent embryos with the stated clonal outcome. Values in parentheses represent the percentage of independent embryos in which the clonal outcome was observed. (iii) Summary of contribution to the biomass of E10.5 yolk sac haematopoietic lineages biomass (based on 5 – 9 element barcodes [58 barcodes]).

c, Induction of barcode formation at E7.5 induction using *Rosa26*iCre (212 barcodes, *n* = 3 embryos). (i) Heatmaps of all informative barcodes. (ii) Biological reproducibility of clonal outcomes. Colours represent the total number of independent embryos with the stated clonal outcome. Values in parentheses represent the percentage of independent embryos in which the clonal outcome was observed. (iii) Summary of contribution to the biomass of E10.5 yolk sac haematopoietic lineages biomass (based on 5 – 9 element barcodes [211 barcodes]).

d, Outcome of *Cdh5*iCre lineage tracking following induction with 4-OHT at E7.5 (*n* = 11 embryos) and E8.5 (*n* = 13 embryos). E10.5 yolk sac lineages (as immunophenotypically defined in Supplementary Fig. 1d – e) were analysed by flow cytometry. Bars, mean ± SEM. Data were analyzed using One-way ANOVA (using Tukey's P value adjustment) was used for multiple comparisons. Exact p-values are shown.

EryP (primitive erythroid), Mk (megakaryocyte), HPC (haematopoietic progenitor/colony forming cell), Mac (Macrophage), Endo (Endothelium).

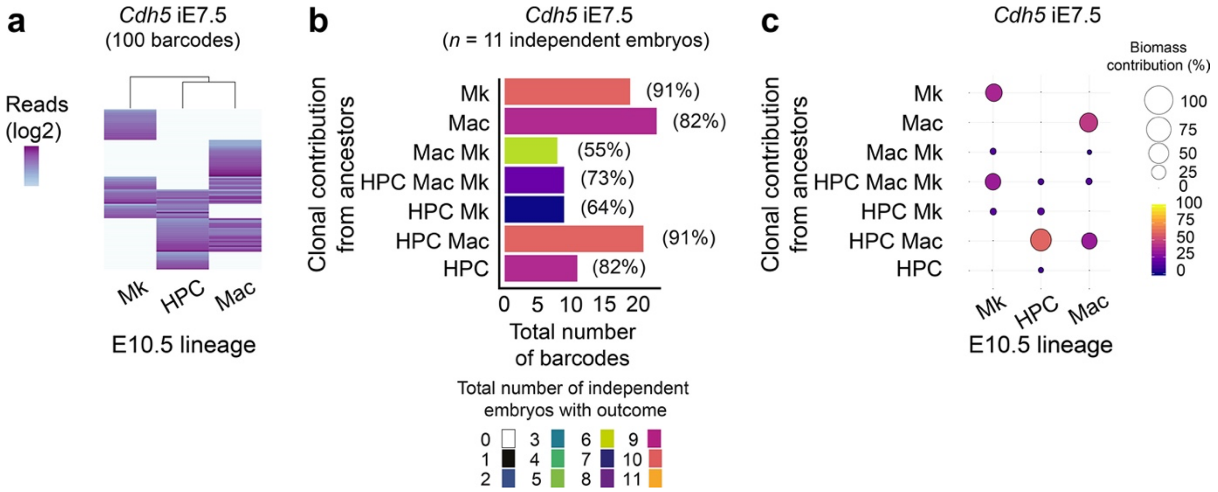

Supplementary Fig. 7: Relationship between non-erythroid haematopoietic lineages.

Induction of barcode formation at E7.5 induction using *Cdh5*iCre.

a, Heatmaps of all informative barcodes (100 barcodes, *n* = 11 independent embryos).

b, Biological reproducibility of clonal outcomes. Colours represent the total number of independent embryos with the stated clonal outcome. Values in parentheses represent the percentage of independent embryos in which the clonal outcome was observed.

c, Summary of contribution to the E10.5 yolk sac non-erythroid haematopoietic lineages biomass (based on 5 – 9 element barcodes [82 barcodes]).

Mk (megakaryocytes), HPC (haematopoietic progenitor/colony forming cell), Mac (macrophage).

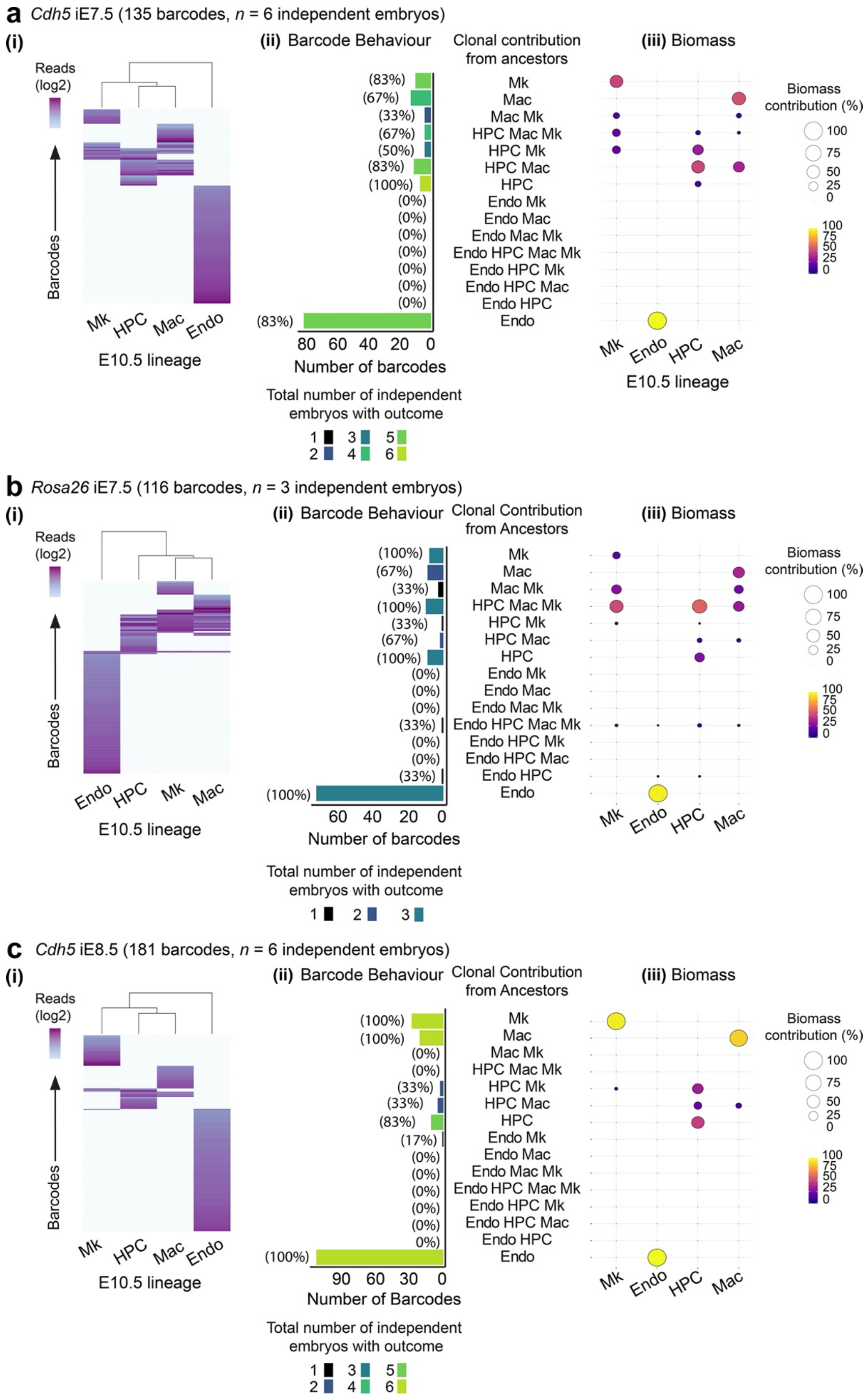

Supplementary Fig. 8: Relationship between the Endo and *Haem* group lineages.

a, Induction of barcode formation at E7.5 using *Cdh5*iCre (135 barcodes, *n* = 6 embryos). **(**i) Heatmaps of all informative barcodes. (ii) Biological reproducibility of clonal outcomes. Colours represent the total number of independent embryos with the stated clonal outcome. Values in parentheses represent the percentage of independent embryos in which the clonal outcome was observed. (iii) Summary of contribution to the E10.5 yolk sac non-erythroid haematopoietic lineages biomass (based on 5 – 9 element barcodes [113 barcodes]).

b, Induction of barcode formation at E7.5 using *Rosa26*iCre (116 barcodes, *n* = 3 embryos). (i) Heatmaps of all informative barcodes. (ii) Biological reproducibility of clonal outcomes. Colours represent the total number of independent embryos with the stated clonal outcome. Values in parentheses represent the percentage of independent embryos in which the clonal outcome was observed. (iii) Summary of contribution to the E10.5 yolk sac non-erythroid haematopoietic lineages biomass (based on 5 – 9 element barcodes [114 barcodes]).

c, Induction of barcode formation at E8.5 using *Cdh5*iCre (181 barcodes, *n* = 6 embryos). (i) Heatmaps of all informative barcodes. (ii) Biological reproducibility of clonal outcomes. Colours represent the total number of independent embryos with the stated clonal outcome. Values in parentheses represent the percentage of independent embryos in which the clonal outcome was observed. (iii) Summary of contribution to the E10.5 yolk sac non-erythroid haematopoietic lineages biomass (based on 5 – 9 element barcodes [148 barcodes]).

Endo (endothelium), Mk (megakaryocytes), HPC (haematopoietic progenitor/colony forming cell), Mac (macrophage).

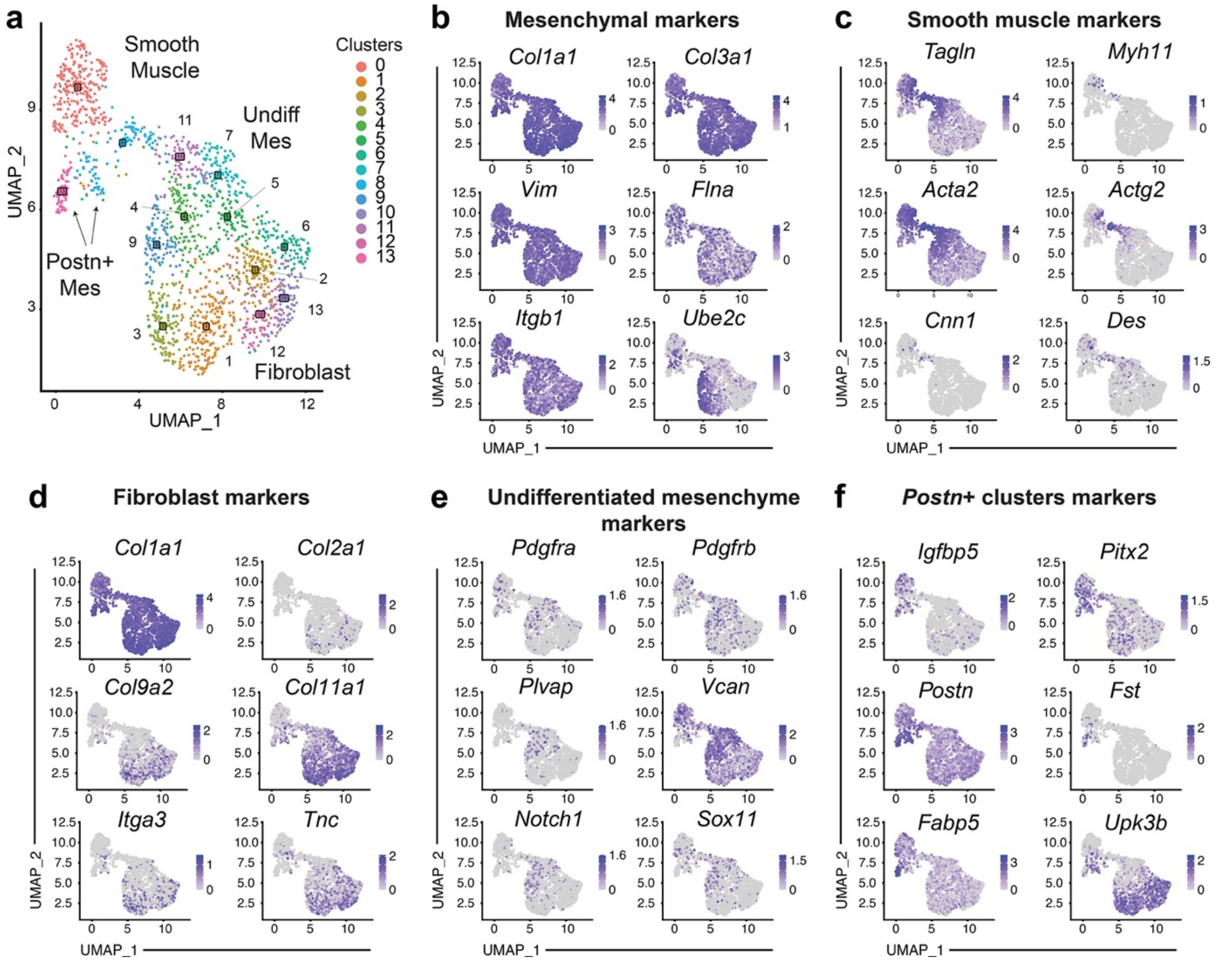

Supplementary Fig. 9: Transcriptional identification of mesenchymal subsets in the E10.5 yolk sac.

a, UMAP with mesenchyme purified from the E10.5 yolk sac. Broad cluster annotations are indicated.

b, Mesenchymal and cell cycle markers expression (*Col1a1, Col3a1, Vim, Flna, Itgb1, Ube2c*).

c, Smooth muscle markers expression (*Tagln, Myh11, Acta2, Actg2, Cnn1, Des*).

d, Fibroblast markers expression (*Col1a1, Col2a1, Col9a2, Col11a1, Itga3, Tnc*).

e, Undifferentiated mesenchyme (Undiff Mesenchyme) markers (*Pdgfra, Pdgfrb, Plvap, Vcan, Notch1, Sox11*).

f, *Postn*+ clusters markers (*Igfbp5, Pitx2, Postn, Fst, Fabp5, Upk3b*).

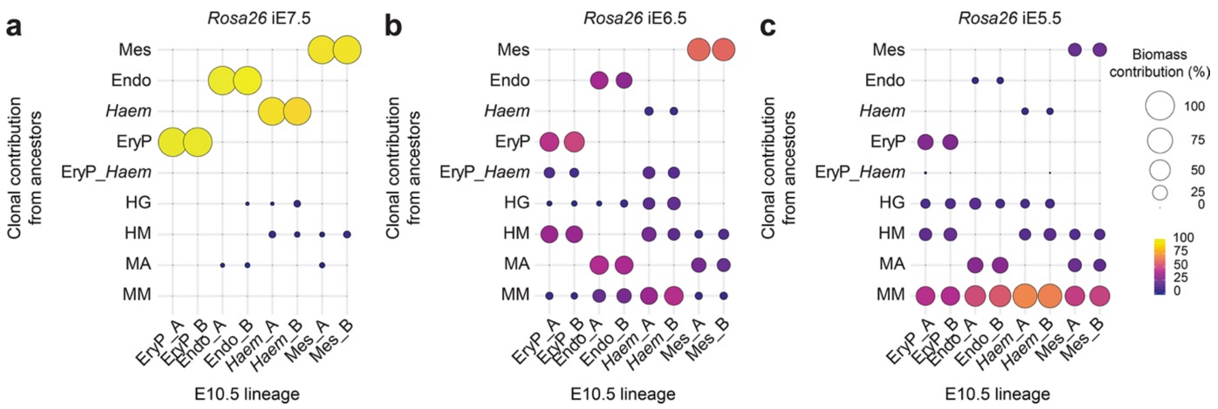

Supplementary Fig. 10: Technical reproducibility of haemangioblast, mesenchymoangioblast, and haematomesoblast outcomes.

a, E7.5 induction using *Rosa26*iCre: summary of technical replicates analyzing contribution to the E10.5 yolk sac non-erythroid haematopoietic lineages biomass. Based on 5 – 9 element barcodes (464 barcodes, *n* = 3 independent embryos).

b, E6.5 induction using *Rosa26*iCre: summary of technical replicates analyzing contribution to the E10.5 yolk sac non-erythroid haematopoietic lineages biomass. Based on 5 – 9 element barcodes (280 barcodes, *n* = 9 independent embryos).

c, E5.5 induction using *Rosa26*iCre: summary of technical replicates analyzing contribution to the E10.5 yolk sac non-erythroid haematopoietic lineages biomass. Based on 5 – 9 element barcodes (163 barcodes, *n* = 14 independent embryos).

Mes (mesenchyme), Endo (endothelium), EryP (primitive erythroid), *Haem* (HPC, megakaryocyte, and macrophage), HG (haemangioblast), HM (haematomesoblast), MA (mesenchymoangioblast), and MM (multi-outcome mesoderm).

Supplementary References

1. Potts KS, Sargeant TJ, Markham JF, et al. A lineage of diploid platelet-forming cells precedes polyploid megakaryocyte formation in the mouse embryo. *Blood*. 2014;124(17):2725-2729.

2. Mikkola HK, Fujiwara Y, Schlaeger TM, Traver D, Orkin SH. Expression of CD41 marks the initiation of definitive hematopoiesis in the mouse embryo. *Blood*. 2003;101(2):508-516.

3. Ferkowicz MJ, Starr M, Xie X, et al. CD41 expression defines the onset of primitive and definitive hematopoiesis in the murine embryo. *Development*. 2003;130(18):4393-4403.

4. Nishikawa SI, Nishikawa S, Kawamoto H, et al. In vitro generation of lymphohematopoietic cells from endothelial cells purified from murine embryos. *Immunity*. 1998;8(6):761-769.

5. Scialdone A, Tanaka Y, Jawaid W, et al. Resolving early mesoderm diversification through single-cell expression profiling. *Nature*. 2016;535(7611):289-293.
