# Supplementary material for: In vivo clonal tracking reveals evidence of haemangioblast and haematomesoblast contribution to yolk sac haematopoiesis
Source: Nat Commun. 2023 Jan 3;14:41. doi: 10.1038/s41467-022-35744-x (PMC9810727; doi:10.1038/s41467-022-35744-x)
Supplement: Supplementary file 2 — Description of Additional Supplementary Files [file 41467_2022_35744_MOESM2_ESM.pdf]

Description of Additional Supplementary Files

File Name: Supplementary Data 1
Description: Antibodies used for flow cytometry/cell sorting.

File Name: Supplementary Data 2
Description: Single-cell RNA-Seq dataset information (Fluidigm).

File Name: Supplementary Data 3
Description: Marker gene identification for E7.25 to E10.5 yolk sac mesodermal populations (Fluidigm dataset). Markers genes were identified using the Scanpy' rank_gene_groups method. Wilcoxon Rank Sum test was used to assess significance of differential gene expression in reference to other clusters. The adjusted p-value was based on Benjamini-Hochberg correction using all genes in the dataset.

File Name: Supplementary Data 4
Description: Estimation of the limit of barcode detection.

File Name: Supplementary Data 5
Description: E10.5 yolk sac lineage LoxCode post-sorting purity analysis.

File Name: Supplementary Data 6
Description: Marker genes for E10.5 yolk sac mesodermal populations (10X dataset). Markers genes were identified using the FindAllMarkers or FindMarkers functions of the Seurat package. Wilcoxon Rank Sum test was used to assess significance of differential gene expression in reference to other clusters. The adjusted p-value was based on Bonferroni correction using all genes in the dataset.

File Name: Supplementary Data 7
Description: Marker genes for E10.5 yolk sac for mesenchymal (Mes) populations (10X dataset). Markers genes were identified using the FindAllMarkers or FindMarkers functions of the Seurat package. Wilcoxon Rank Sum test was used to assess significance of differential gene expression in reference to other clusters. The adjusted p-value was based on Bonferroni correction using all genes in the dataset.

File Name: Supplementary Data 8
Description: Numbers of LoxCode barcode detected in experimental groups according to Cre-driving mouse line, developmental stage of induction, and E10.5 lineages investigated.

File Name: Supplementary Software 1
Description: Interactive 3D UMAP of single-cell RNA-Seq data with cells coloured according to lineage immunophenotype (html file that can viewed in a web browser).

File Name: Supplementary Software 2
Description: Interactive hybrid 3D UMAP of single-cell RNA-Seq data coloured according to lineage immunophenotype or transcriptional cluster (html file that can viewed in a web browser).
