# Supplementary material for: In vivo clonal tracking reveals evidence of haemangioblast and haematomesoblast contribution to yolk sac haematopoiesis
Source: Nat Commun. 2023 Jan 3;14:41. doi: 10.1038/s41467-022-35744-x (PMC9810727; doi:10.1038/s41467-022-35744-x)
Supplement: Supplementary file 13 — Reporting Summary [file 41467_2022_35744_MOESM13_ESM.pdf]

nature research

Corresponding author(s): Samir Taoudi
Shalin Naik

Last updated by author(s): 12/19/2022

Reporting Summary

Nature Research wishes to improve the reproducibility of the work that we publish. This form provides structure for consistency and transparency in reporting. For further information on Nature Research policies, see our Editorial Policies and the Editorial Policy Checklist.

Statistics

For all statistical analyses, confirm that the following items are present in the figure legend, table legend, main text, or Methods section.

n/a	Confirmed	
☐	☒	The exact sample size (*n*) for each experimental group/condition, given as a discrete number and unit of measurement
☐	☒	A statement on whether measurements were taken from distinct samples or whether the same sample was measured repeatedly
☐	☒	The statistical test(s) used AND whether they are one- or two-sided<br>*Only common tests should be described solely by name; describe more complex techniques in the Methods section.*
☒	☐	A description of all covariates tested
☒	☐	A description of any assumptions or corrections, such as tests of normality and adjustment for multiple comparisons
☒	☐	A full description of the statistical parameters including central tendency (e.g. means) or other basic estimates (e.g. regression coefficient) AND variation (e.g. standard deviation) or associated estimates of uncertainty (e.g. confidence intervals)
☒	☐	For null hypothesis testing, the test statistic (e.g. *F*, *t*, *r*) with confidence intervals, effect sizes, degrees of freedom and *P* value noted<br>*Give P values as exact values whenever suitable.*
☒	☐	For Bayesian analysis, information on the choice of priors and Markov chain Monte Carlo settings
☒	☐	For hierarchical and complex designs, identification of the appropriate level for tests and full reporting of outcomes
☒	☐	Estimates of effect sizes (e.g. Cohen's *d*, Pearson's *r*), indicating how they were calculated

Our web collection on statistics for biologists contains articles on many of the points above.

Software and code

Policy information about availability of computer code

Data collection	Sequencing data:<br>- bc2fastq (Illumina) to make the fastq files<br>- custom LoxCode code for extracting the barcodes from the raw data and making the .rds files (LoxCodes, number of reads, first detection, barcode length, barcode complexity, metadata re-sample)<br>- custom R code to extract data from the .rds file and analyse barcode distribution and reads.
Data analysis	Single cell RNA sequencing dataset: The fastq files were aligned to Ensembl mouse genome version 84 using Rsubread package (doi:10.18129/B9.bioc.Rsubread). The featureCounts function from the same package was used to generate counts matrix summarised at the gene level. Starting from 1073 samples at this stage, the scater package (doi:10.18129/B9.bioc.scater) in R was then used to filter out outlier samples and genes to produce a matrix with 926 samples and 15,967 genes. Then the scran package (doi:10.18129/B9.bioc.scran) from R was used to normalise the matrix using computeSumFactors function, and cpm values were generated using calculateCPM function. Multiple dimensionality reduction techniques were applied in order to check that results were robust - including TSNE, PCA, UMAP and more, across a range of parameters. The results shown here were obtained using the python umap package, with n_neighbors=40 and min_dist=0.8. K-means clustering was used to create 20 clusters from the UMAP projection in 3 dimensions. Differentially expressed genes between relevant clusters were obtained using edgeR package (doi:10.18129/B9.bioc.edgeR) in R. GO terms analysis was carried out using PANTHER (http://geneontology.org/page/go-enrichment-analysis). Heatmaps were generated using Morpheus software (https://software.broadinstitute.org/morpheus/).<br><br>Single cell 10X RNA sequencing dataset: CellRanger was used to extract raw data. Transcriptomes were scaled using ScTransform. Seurat clusters were identified and annotated using DE gene lists. A high quality 7,316 cells dataset was obtained after screening for transcriptome quality (> 3000 UMI, > 1000 features, < 5 % mitochondrial transcripts) and excluding cells with inconclusive hashtag calls or multiplets. Differential expression analysis in Fig 5, Sfig 3 and 9 was carried out using Seurat.

LoxCodes analysis (custom R code): once LoxCodes were extracted, they were subject to filtering (detection threshold, removal of barcodes that did not fit the expected structure, and removal of PCR artefacts) as described in material and methods and Sfig.5. Control experiments with various clones containing known barcodes were used to assess the fidelity of PCR and the linearity between reads and cell input. In these experiments, low level barcode breakdown during PCR was noted (SFig.4C). These experiments were used as a template to remove potential PCR artefacts: for each barcode (eg 1 2 7), the reads of all barcodes that contained its constituting elements in the same or opposite orientation (eg 1 2 7 13, 1 2 4 -7 etc..) were tallied. the tested barcode was removed if its own reads represented less than 10% of the reads of combined potential parents. In control experiments, this removed all background (SFig.4C). In addition, due to the possibility of independent formation of the same barcode in 2 different cells (which could lead to spurious connections), classes of barcodes rare enough to be most likely clonal were identified using their size and complexity (SFig5). Unique barcodes belonging to classes with >2000 combinatorial possibilities and detected in one embryo only (informative barcodes) were selected for further analysis. Legitimate barcodes samples were then normalised for the number of reads/cell for each population. The results were displayed using Heatmap2.0, bar and bubble plots (ggplot2).

For manuscripts utilizing custom algorithms or software that are central to the research but not yet described in published literature, software must be made available to editors and reviewers. We strongly encourage code deposition in a community repository (e.g. GitHub). See the Nature Research guidelines for submitting code & software for further information.

Data

Policy information about availability of data

All manuscripts must include a data availability statement. This statement should provide the following information, where applicable:

- Accession codes, unique identifiers, or web links for publicly available datasets
- A list of figures that have associated raw data
- A description of any restrictions on data availability

Single cell RNA sequencing data has been made available through NCBI's Gene Expression Omnibus
Fluidigm dataset:
accession GSE164336. The GSE164336 dataset can be accessed by entering the token "mvybmosejjqjreb" at:
"https://protect-au.mimecast.com/s/0gaZCVAGLrsxO16O4hG9UHJ?domain=ncbi.nlm.nih.gov".

10X dataset: accession GSE204896. The GSE204896 dataset can be accessed by entering the token "ezirqwckdnklxqf" at:
https://www.ncbi.nlm.nih.gov/geo/query/acc.cgi?acc=GSE204896.

Computer code will be available upon reasonable request.

Field-specific reporting

Please select the one below that is the best fit for your research. If you are not sure, read the appropriate sections before making your selection.

☒ Life sciences ☐ Behavioural & social sciences ☐ Ecological, evolutionary & environmental sciences

For a reference copy of the document with all sections, see nature.com/documents/nr-reporting-summary-flat.pdf

Life sciences study design

All studies must disclose on these points even when the disclosure is negative.

Sample size	>=3 embryos were used per experiment. Embryo numbers are indicated in the figure legends for each experiment.
Data exclusions	Embryos that looked unhealthy or were too young or too old (>0.5 day in either direction) for the representative stage were excluded.
Replication	>=3 embryos were used per experiment.
Randomization	Embryonic samples were allocated to a given group depending on the experimental design followed to generate them and the stage of the embryo at the time of collection.
Blinding	Other than the stage and state of health, embryonic samples were treated as is.

Reporting for specific materials, systems and methods

We require information from authors about some types of materials, experimental systems and methods used in many studies. Here, indicate whether each material, system or method listed is relevant to your study. If you are not sure if a list item applies to your research, read the appropriate section before selecting a response.

Materials & experimental systems

n/a	Involved in the study
☐	☒ Antibodies
☐	☐ Eukaryotic cell lines
☐	☐ Palaeontology and archaeology
☐	☒ Animals and other organisms
☐	☐ Human research participants
☐	☐ Clinical data
☐	☐ Dual use research of concern

Methods

n/a	Involved in the study
☐	☐ ChIP-seq
☐	☒ Flow cytometry
☐	☐ MRI-based neuroimaging

Antibodies

Antibodies used

Biolegend (PDGFRA (APA5, BV605, #135916), CD31/PECAM (390, BV421, #102424), CDH5 (BV13, Alexa647, #138006), CX3CR1 (SA011F11, BV650, #149033)), eBiosciences (CD41 (eBioMWReg30, PE, #12-0411-82)) or made in house (Ter119 (Ter119), CD45 (30F11, APC), F4/80 (BM8, Alexa700)). Specificity for each antibody is indicated on the technical data sheets. Antibodies list can be found in Table S1.

Validation

All antibodies are well characterised antibodies, broadly used in scientific publications (PDGFRA, APA5 clone, 5 publications; CD41, eBioMWReg30 clone, 20 publications; CD31/PECAM, 390 clone, >20 publications; Ter119 clone, 723 publications; CD45, 30F11 clone, 11 publications; F4/80: 807 publications; CX3CR1: 340 publications, CDH5: 2 publications). Haematopoietic and endothelial populations isolated with combinations of these antibodies have been formally characterised in previous publications from our laboratory (referenced in the paper) and single cell sequenced in this work, which confirmed their identity.

Eukaryotic cell lines

Policy information about cell lines

Cell line source(s)

State the source of each cell line used.

Authentication

Describe the authentication procedures for each cell line used OR declare that none of the cell lines used were authenticated.

Mycoplasma contamination

Confirm that all cell lines tested negative for mycoplasma contamination OR describe the results of the testing for mycoplasma contamination OR declare that the cell lines were not tested for mycoplasma contamination.

Commonly misidentified lines
(See ICLAC register)

Name any commonly misidentified cell lines used in the study and provide a rationale for their use.

Palaeontology and Archaeology

Specimen provenance

Provide provenance information for specimens and describe permits that were obtained for the work (including the name of the issuing authority, the date of issue, and any identifying information).

Specimen deposition

Indicate where the specimens have been deposited to permit free access by other researchers.

Dating methods

If new dates are provided, describe how they were obtained (e.g. collection, storage, sample pretreatment and measurement), where they were obtained (i.e. lab name), the calibration program and the protocol for quality assurance OR state that no new dates are provided.

☐ Tick this box to confirm that the raw and calibrated dates are available in the paper or in Supplementary Information.

Ethics oversight

Identify the organization(s) that approved or provided guidance on the study protocol, OR state that no ethical approval or guidance was required and explain why not.

Note that full information on the approval of the study protocol must also be provided in the manuscript.

Animals and other organisms

Policy information about studies involving animals; ARRIVE guidelines recommended for reporting animal research

Laboratory animals

All mouse lines were maintained on a C57BL/6 background. Adult (2-9 months old) males and females were bred to generate embryos.

Wild animals

n/a

Field-collected samples

For laboratory work with field-collected samples, describe all relevant parameters such as housing, maintenance, temperature, photoperiod and end-of-experiment protocol OR state that the study did not involve samples collected from the field.

Ethics oversight	Experiments were covered by the Walter and Eliza Hall Institute Animal Ethics Committee Applications # 2014.025, 2017.39, 2018.015, 2020.051.

Note that full information on the approval of the study protocol must also be provided in the manuscript.

Human research participants

Policy information about studies involving human research participants

Population characteristics	*Describe the covariate-relevant population characteristics of the human research participants (e.g. age, gender, genotypic information, past and current diagnosis and treatment categories). If you filled out the behavioural & social sciences study design questions and have nothing to add here, write "See above."*
Recruitment	*Describe how participants were recruited. Outline any potential self-selection bias or other biases that may be present and how these are likely to impact results.*
Ethics oversight	*Identify the organization(s) that approved the study protocol.*

Note that full information on the approval of the study protocol must also be provided in the manuscript.

Clinical data

Policy information about clinical studies

All manuscripts should comply with the ICMJE guidelines for publication of clinical research and a completed CONSORT checklist must be included with all submissions.

Clinical trial registration	*Provide the trial registration number from ClinicalTrials.gov or an equivalent agency.*
Study protocol	*Note where the full trial protocol can be accessed OR if not available, explain why.*
Data collection	*Describe the settings and locales of data collection, noting the time periods of recruitment and data collection.*
Outcomes	*Describe how you pre-defined primary and secondary outcome measures and how you assessed these measures.*

Dual use research of concern

Policy information about dual use research of concern

Hazards

Could the accidental, deliberate or reckless misuse of agents or technologies generated in the work, or the application of information presented in the manuscript, pose a threat to:

No	Yes	
☒	☐	Public health
☒	☐	National security
☒	☐	Crops and/or livestock
☒	☐	Ecosystems
☒	☐	Any other significant area

Experiments of concern

Does the work involve any of these experiments of concern:

No	Yes	
☒	☐	Demonstrate how to render a vaccine ineffective
☒	☐	Confer resistance to therapeutically useful antibiotics or antiviral agents
☒	☐	Enhance the virulence of a pathogen or render a nonpathogen virulent
☒	☐	Increase transmissibility of a pathogen
☒	☐	Alter the host range of a pathogen
☒	☐	Enable evasion of diagnostic/detection modalities
☒	☐	Enable the weaponization of a biological agent or toxin
☒	☐	Any other potentially harmful combination of experiments and agents

ChIP-seq

Data deposition

☐ Confirm that both raw and final processed data have been deposited in a public database such as GEO.

☐ Confirm that you have deposited or provided access to graph files (e.g. BED files) for the called peaks.

Data access links<br>*May remain private before publication.*	*For "Initial submission" or "Revised version" documents, provide reviewer access links. For your "Final submission" document, provide a link to the deposited data.*
Files in database submission	*Provide a list of all files available in the database submission.*
Genome browser session<br>(e.g. UCSC)	*Provide a link to an anonymized genome browser session for "Initial submission" and "Revised version" documents only, to enable peer review. Write "no longer applicable" for "Final submission" documents.*

Methodology

Replicates	*Describe the experimental replicates, specifying number, type and replicate agreement.*
Sequencing depth	*Describe the sequencing depth for each experiment, providing the total number of reads, uniquely mapped reads, length of reads and whether they were paired- or single-end.*
Antibodies	*Describe the antibodies used for the ChIP-seq experiments; as applicable, provide supplier name, catalog number, clone name, and lot number.*
Peak calling parameters	*Specify the command line program and parameters used for read mapping and peak calling, including the ChIP, control and index files used.*
Data quality	*Describe the methods used to ensure data quality in full detail, including how many peaks are at FDR 5% and above 5-fold enrichment.*
Software	*Describe the software used to collect and analyze the ChIP-seq data. For custom code that has been deposited into a community repository, provide accession details.*

Flow Cytometry

Plots

Confirm that:

☐ The axis labels state the marker and fluorochrome used (e.g. CD4-FITC).

☐ The axis scales are clearly visible. Include numbers along axes only for bottom left plot of group (a 'group' is an analysis of identical markers).

☒ All plots are contour plots with outliers or pseudocolor plots.

☐ A numerical value for number of cells or percentage (with statistics) is provided.

Methodology

Sample preparation	All cells came from mouse embryos E7.5-E10.5 bred as described above. Extraembryonic samples were enzymatically dissociated, filtered, labelled with antibodies and analysed by flow cytometry.
Instrument	Fortessa II
Software	FlowJo 7 to 10 (depending when the experiments were run).
Cell population abundance	All sorted samples were purity checked. 5-20% of the sample was re-run depending on abundance. Average purity was 98%. Purity data are included in Table S5.
Gating strategy	Gating strategies are shown in the paper (immunophenotype disclosed for previously published populations, from the live single cell gate for the others). Single live cells were selected by removing low FSC and high SSC cells and excluding cells curving away from the diagonal on both FSC_A/FSC_H and SSC_A/SSC_H plots. 7AAD was used to discard dead cells.

☒ Tick this box to confirm that a figure exemplifying the gating strategy is provided in the Supplementary Information.

Magnetic resonance imaging

Experimental design

Design type	*Indicate task or resting state; event-related or block design.*

Design specifications

Specify the number of blocks, trials or experimental units per session and/or subject, and specify the length of each trial or block (if trials are blocked) and interval between trials.

Behavioral performance measures

State number and/or type of variables recorded (e.g. correct button press, response time) and what statistics were used to establish that the subjects were performing the task as expected (e.g. mean, range, and/or standard deviation across subjects).

Acquisition

Imaging type(s)

Specify: functional, structural, diffusion, perfusion.

Field strength

Specify in Tesla

Sequence & imaging parameters

Specify the pulse sequence type (gradient echo, spin echo, etc.), imaging type (EPI, spiral, etc.), field of view, matrix size, slice thickness, orientation and TE/TR/flip angle.

Area of acquisition

State whether a whole brain scan was used OR define the area of acquisition, describing how the region was determined.

Diffusion MRI ☐ Used ☐ Not used

Preprocessing

Preprocessing software

Provide detail on software version and revision number and on specific parameters (model/functions, brain extraction, segmentation, smoothing kernel size, etc.).

Normalization

If data were normalized/standardized, describe the approach(es): specify linear or non-linear and define image types used for transformation OR indicate that data were not normalized and explain rationale for lack of normalization.

Normalization template

Describe the template used for normalization/transformation, specifying subject space or group standardized space (e.g. original Talairach, MNI305, ICBM152) OR indicate that the data were not normalized.

Noise and artifact removal

Describe your procedure(s) for artifact and structured noise removal, specifying motion parameters, tissue signals and physiological signals (heart rate, respiration).

Volume censoring

Define your software and/or method and criteria for volume censoring, and state the extent of such censoring.

Statistical modeling & inference

Model type and settings

Specify type (mass univariate, multivariate, RSA, predictive, etc.) and describe essential details of the model at the first and second levels (e.g. fixed, random or mixed effects; drift or auto-correlation).

Effect(s) tested

Define precise effect in terms of the task or stimulus conditions instead of psychological concepts and indicate whether ANOVA or factorial designs were used.

Specify type of analysis: ☐ Whole brain ☐ ROI-based ☐ Both

Statistic type for inference
(See Eklund et al. 2016)

Specify voxel-wise or cluster-wise and report all relevant parameters for cluster-wise methods.

Correction

Describe the type of correction and how it is obtained for multiple comparisons (e.g. FWE, FDR, permutation or Monte Carlo).

Models & analysis

n/a	Involved in the study
☐	☐ Functional and/or effective connectivity
☐	☐ Graph analysis
☐	☐ Multivariate modeling or predictive analysis

Functional and/or effective connectivity

Report the measures of dependence used and the model details (e.g. Pearson correlation, partial correlation, mutual information).

Graph analysis

Report the dependent variable and connectivity measure, specifying weighted graph or binarized graph, subject- or group-level, and the global and/or node summaries used (e.g. clustering coefficient, efficiency, etc.).

Multivariate modeling and predictive analysis

Specify independent variables, features extraction and dimension reduction, model, training and evaluation metrics.
